# A Metal‐Free Molecular Ferroelectric With Large Piezoelectricity and Its Porous Composite for Superior Output Power Density

**DOI:** 10.1002/advs.202524032

**Published:** 2026-03-02

**Authors:** Nan Chen, Wenjuan Wei, Le Ye, Lingyu Wan, Yen Wei

**Affiliations:** ^1^ Center on Nanoenergy Research School of Physical Science and Technology Guangxi University Nanning P. R. China; ^2^ Department of Chemistry Tsinghua University Beijing P. R. China; ^3^ Department of Chemistry Southern University of Science and Technology Shenzhen Guangdong P. R. China

**Keywords:** metal‐free molecular ferroelectric, piezoelectricity, piezoelectric energy harvesting, superior output power density, untrasonic detection

## Abstract

The key to achieving high power density in piezoelectric materials lies in the synergistic optimization of both high piezoelectric constant (*d*_33_) and piezoelectric voltage coefficient (*g*_33_). Traditional inorganic ceramics offer high *d*_33_ but suffer from low *g*_33_, poor flexibility, and toxic‐Pb content. Polymers, while flexible, are limited by their relatively low *d*_33_ and insufficient stability. Molecular ferroelectrics can simultaneously offer high piezoelectricity, flexibility, and low loss, yet most reported systems contain toxic‐Cd components, restricting their use in biomedical and wearable applications. Metal‐free molecular ferroelectrics, replaced toxic metals while retaining the molecular ferroelectric attributes, combine environmental friendliness, and biocompatibility, making them ideal candidates for piezoelectrics. However, their piezoelectric output has traditionally been weak. Here, a novel metal‐free molecular ferroelectric, [(CH_3_C_6_H_10_NH_3_)(18‐crown‐6)][ClO_4_] (CH_3_C_6_H_10_NH_3_ = trans‐4‐methylcyclohexylaminium), was designed and synthesized via a methylation‐assisted ring‐expansion strategy, enhancing the piezoelectric performance. The material exhibits a *d*_33_ of 67.42 ± 1.87 pC/N and a *g*_33_ of approximately 2768.9 × 10^−3^ V m/N. When composited with thermoplastic polyurethane (TPU) into a porous architecture, the material delivers an output voltage of 120 V and a power density of 432.1 µW/cm^2^, representing the highest performance reported among metal‐free molecular piezoelectrics. This work offers a practical way to design high‐performance, biocompatible piezoelectrics, creating a new platform for flexible sensors and self‐powered devices.

## Graphical abstract

Metal‐free molecular crystal [(CH_3_C_6_H_10_NH_3_)(18‐crown‐6)][ClO_4_] functions as a high‐performance molecular ferroelectric. Through strategic methylation of the cyclohexylamine unit, the compound achieves large spontaneous polarization and outstanding piezoelectric coefficients. When incorporated into flexible porous composites, the material delivers exceptional piezoelectric energy‐harvesting outputs, attaining a record power density among all‐organic molecular ferroelectrics. These attributes enable its effective use in advanced applications such as underwater ultrasonic detection.

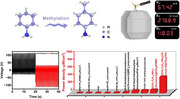

## 1 Introduction

The essence of the high output power density of piezoelectrics lies in the realization of efficient energy conversion with a small volume and low input energy, which directly drives the development of miniaturized, passive, and intelligent electronic systems. It not only addresses the core challenges of limited endurance and cumbersome maintenance for traditional batteries, but also unlocks a vast array of self‐powered application scenarios spanning from wearable devices to industrial monitoring, thus serving as a pivotal breakthrough in cutting‐edge energy harvesting technologies. Achieving a high power density requires the balanced optimization of a high piezoelectric coefficient *d*_33_ (which ensures charge generation) and a high piezoelectric voltage coefficient *g*_33_ (which guarantees voltage output). These two parameters are generally recognized as the key metrics for evaluating the performance of piezoelectrics, and they respectively reflect the actuation performance and sensing sensitivity of such materials. Optimization of the intrinsic material properties underpins electromechanical energy conversion. Ferroelectrics, the core of piezoelectrics, feature intrinsic reversible ferroelectric polarization that serves as the macroscopic origin of the piezoelectric effect through external field‐induced polarization deformation [1, 2, 3, 4]. They show irreplaceable advantages in piezoelectric performance upper limit, tunability, and energy conversion efficiency [5, 6, 7, 8]. For decades, extensively studied materials such as inorganic ferroelectric ceramics and organic ferroelectric polymers have demonstrated significant potential for piezoelectric applications [9, 10, 11]. Ceramics like lead titanate (PbTiO_3_) and its zirconium‐doped derivative, lead zirconate titanate Pb(Zr_0.52_Ti_0.48_)O_3_ (PZT), offer strong piezoelectric *d*_33_ [12, 13], but they are stiff, contain toxic metals, and require complex processing [9, 10]. Their high dielectric constant (ε_*r*_) also leads to a low *g*_33_, making them less suitable for flexible electronics. In contrast, polymers like PVDF and PVDF‐TrFE are flexible and easier to process. Although their *d*_33_ (‐20 ∼ ‐40 pC/N) is lower, they have a higher *g*_33_ (∼237–310 × 10^−3^ V m/N) thanks to their low ε_*r*_ [14, 15]. However, they suffer from poor stability and limited crystallinity, which restricts their use in demanding applications [11].

Abandon the performance trade‐off of traditional inorganic ceramics/polymers, and develop new systems with low *ε*_r_, high *d*_33_, *g*_33_, and low loss to break through the upper limit of power density from the source. State‐of‐the‐art molecular ferroelectrics break conventional performance trade‐offs, realize synergy of high piezoelectric performance, flexibility, and low loss, and serve as core materials for high‐power‐density, flexible, lead‐free, and miniaturized piezoelectric devices, which have great practical value in ultra‐precision instruments, medical ultrasound, and acoustic/underwater detection [2, 4, 16]. Additionally, the relatively weak intermolecular forces in molecular ferroelectrics enable a balance between rigidity and toughness. This results in a low elastic modulus, allowing the material to maintain flexibility under mechanical stress and thereby broadening its application potential in fields such as flexible electronics. To date, many molecular ferroelectrics have shown excellent piezoelectric performance. For example, TMCM‐CdCl_3_ (TMCM = trimethyl‐chloromethylammonium) achieves a *d*_33_ of 220 pC/N [17], while its modified version TMCM‐CdBrCl_2_ reaches 440 pC/N, with a high *g*_33_ of 6215 × 10^−3^ V·m/N [18]. The most notable is (TMFM)_0.26_(TMCM)_0.74_CdCl_3_, which attains a remarkably high *d*_33_ of 1540 pC/N [19]. These materials are promising for use in flexible sensors and energy harvesters. Recent developments include TMCM‐CdCl_3_/PDMS films that generate 41 V voltage output, 3.45 µA/cm^2^ current, and a power density of 115.2 µW/cm^2^ with high *d*_33_ of 61.3 pC/N and *g*_33_ of 2590 × 10^−3^ V m/N [20, 21, 22], and TMCM‐CdCl_3_/TPU composites that achieve 103 V, 42 µA and 636.9 µW/cm^2^ [23]. However, these materials still contain toxic metal Cd, which limits their use in medical and flexible electronics.

Metal‐free molecular ferroelectrics, composed entirely of organic building blocks, offer a promising alternative to conventional metal‐containing systems due to their inherent environmental friendliness and biocompatibility. Their organic nature imparts structural flexibility, low weight, and a low dielectric constant, while their polar noncentrosymmetric structures enable rich physical effects such as piezoelectricity [24, 25]. These characteristics make them particularly suitable for applications requiring high *g*_33_. The structural versatility of organic molecules allows for tailored design through chemical modification, which can optimize electromechanical performance. For example, the fluorinated two‐imine‐linked 2D covalent organic frameworks have achieved *d*_33_ values of 20.9 and 18.9 pC/N [26], while a fluorinated crown‐ether‐based molecular ferroelectric exhibits a *d*_33_ of 42 pC/N and a *g*_33_ of 680 × 10^−3^ V m/N) [27]. In addition, ring expansion strategies have been employed to reduce molecular strain, such as in [3.2.1‐abco]ReO_4_, which achieves a *d*_33_ of ∼118 pC/N [28]. However, while metal‐free alternatives are more sustainable, their piezoelectric output has traditionally been weaker, making the development of high‐performance metal‐free ferroelectrics a key research challenge [29, 30].

Building on these approaches, we have designed a novel metal‐free molecular ferroelectric, [(CH_3_C_6_H_10_‐NH_3_)(18‐crown‐6)][ClO_4_] (CH_3_‐C_6_H_10_‐NH_3_ = trans‐4‐methylcyclohexylaminium), through methyl‐assisted ring expansion of a known cyclohexylaminium‐based system [31]. This compound demonstrates excellent ferroelectric and piezoelectric properties, with a *d*_33_ of 67.42 ± 1.87 pC/N and a high *g*_33_ of approximately 2768.9 × 10^−3^ V·m/N. Moreover, its strong electromechanical coupling makes it highly suitable for energy harvesting and sensing. When integrated into a porous thermoplastic polyurethane (TPU) composite, the material delivers a remarkable piezoelectric output of 120 V and a power density of 432.1 µW/cm^2^, which is undoubtedly the highest among the metal‐free molecular piezoelectrics. These results not only demonstrate the potential of rational molecular design in developing high‐performance metal‐free piezoelectrics but also highlight their viability for next‐generation bio‐compatible sensing and energy‐harvesting technologies.

## 2 Results and Discussion

**FIGURE 1:**
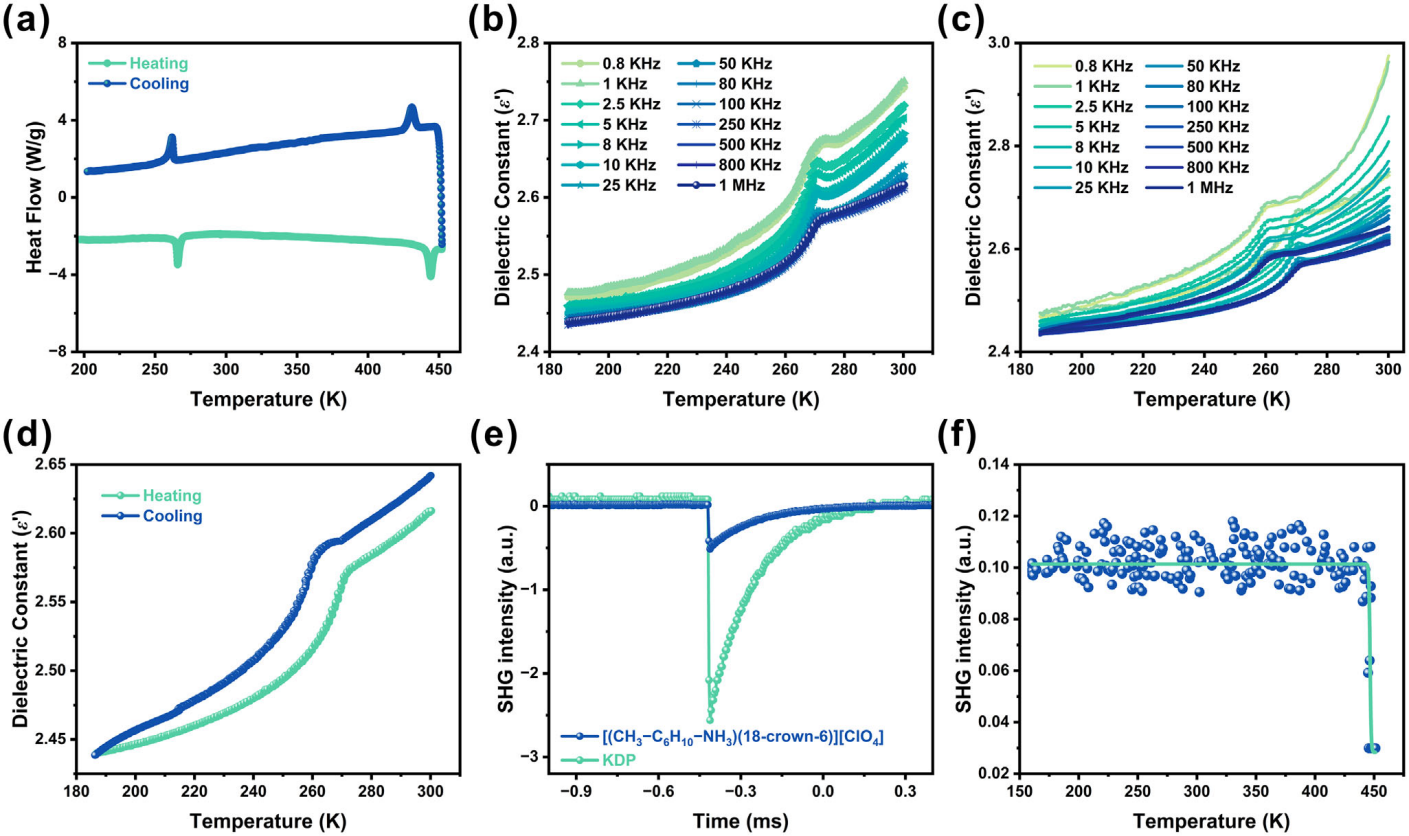
(a) DSC curves. (b) the real part (*ε'*) of dielectric permittivity during the heating process. (c) Temperature‐dependent *ε'* during cooling‐heating cycles. (d) Temperature‐dependent ε′ at 1 MHz. (e) SHG signals for the synthesized crystals and KDP at room temperature. (f) Temperature‐dependent SHG response of [(CH_3_‐C_6_H_10_‐NH_3_)(18‐crown‐6)][ClO_4_] crystals in the heating process.

The block‐like crystals of [(CH_3_C_6_H_10_NH_3_)(18‐crown‐6)][ClO_4_] were obtained by slow evaporation of a methanol solution containing stoichiometric *trans*‐4‐methylcyclohexylamine, 18‐crown‐6, and perchloric acid. These crystals exhibit good thermal stability up to 452 K (Figure S1), and their phase purity was confirmed by room‐temperature powder X‐ray diffraction (PXRD, Figure S2). Differential scanning calorimetry (DSC) reveals two reversible thermal anomalies during heating/cooling cycles: a solid‐solid transition at *T*_1_ = 266/262 K and a solid‐liquid transition at the melting point *T*_2_ = 444/432 K (Figure 1a). Accordingly, the phases below *T*_1_, between *T*_1_ and *T*_2_, and above *T*_2_ are denoted as the low‐temperature (LTP), intermediate (ITP), and high‐temperature (HTP) phases, respectively. Compared with the non‐methylated analogue [(C_6_H_11_NH_3_)(18‐crown‐6)][ClO_4_] [31], the methylated compound undergoes two distinct phase transitions and remains in a ferroelectric state up to its melting point. This modification raises the Curie temperature (*T*_c_) by 54 K, resulting in a *T*_c_ comparable to the highest‐*T*_c_ crown‐ether‐based molecular ferroelectrics (∼450 K) [32] and thus enabling a wide operational temperature window. Dielectric measurements (185–300 K, 0.8 kHz–1 MHz) further confirm the reversible phase transition. The real permittivity (*ε*′) displays a λ‐shaped anomaly near *T*_1_ without frequency dispersion, while the magnitude of *ε*′ decreases gradually with increasing frequency (Figure 1b). A step‐like dielectric switching is observed during heating‐cooling cycles, with *ε*′ ≈ 2.62 at 1 MHz near room temperature (Figure 1c,d). These features are characteristic of a second‐order ferroelectric phase transition, corresponding to an ordered‐to‐disordered structural transformation. Second‐harmonic generation (SHG) measurements show that the crystals exhibit non‐centrosymmetric structure at room temperature, with an SHG intensity ∼0.2 × KDP (Figure 1c; Figure S3). The SHG signal remains active until *T*_2_, where it abruptly drops to zero, confirming a noncentrosymmetric to centrosymmetric transition at the melting point (Figure 1d).

**FIGURE 2:**
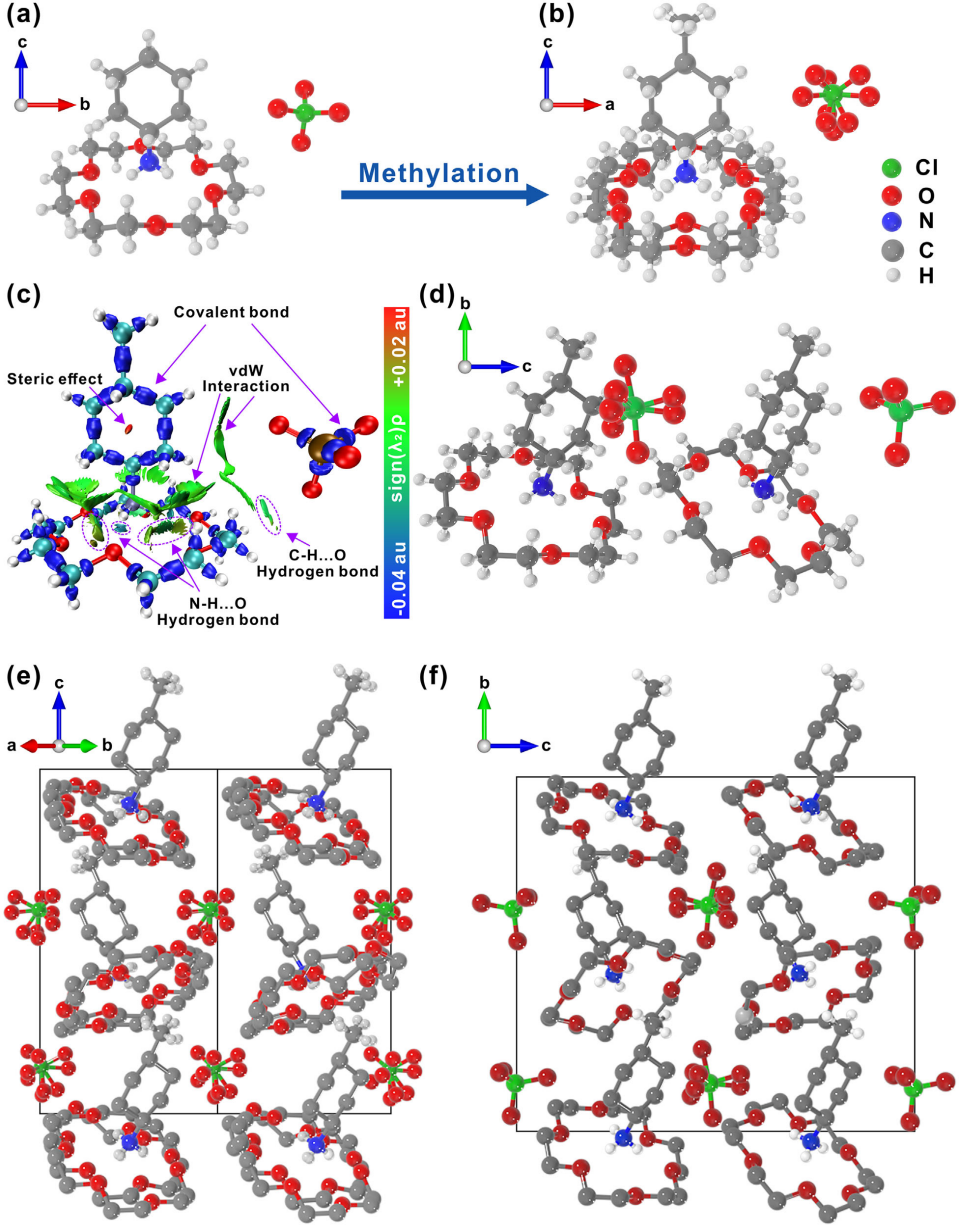
The basic structures of (a) [(C_6_H_11_NH_3_)(18‐crown‐6)][ClO_4_] at room temperature and (b) [(CH_3_‐C_6_H_10_‐NH_3_)(18‐crown‐6)][ClO_4_] in ITP. (c) Isosurface map of [(CH_3_‐C_6_H_10_‐NH_3_)(18‐crown‐6)][ClO_4_], showing hydrogen bonding interactions. (d) The basic structures of [(CH_3_‐C_6_H_10_‐NH_3_)(18‐crown‐6)][ClO_4_] in LTP. Packing view of structures of [(CH_3_‐C_6_H_10_‐NH_3_)(18‐crown‐6)][ClO_4_] in (e) ITP and (f) LTP. Parts of hydrogen atoms were omitted for clarity.

Crystal structures of [(CH_3_C_6_H_10_NH_3_)(18‐crown‐6)][ClO_4_] and its non‐methylated analogue [(C_6_H_11_NH_3_)(18‐crown‐6)][ClO_4_] were determined by variable‐temperature single‐crystal X‐ray diffraction. At room temperature (293 K, ITP), both compounds crystallize in orthorhombic space groups (*Cmc*2_1_ and *Pca*2_1_, respectively) with point group *mm*2. In each structure, the protonated cyclohexylamine units are anchored within the cavity of the 18‐crown‐6 macrocycle via strong N‐H···O hydrogen bonds and adopt a zigzag packing arrangement (Figure 2; Figure S4) [31]. The asymmetric unit of the methylated compound contains half a *trans*‐4‐methylcyclohexylammonium cation, half an 18‐crown‐6 molecule, and half a ClO_4_^−^ anion, whereas that of the non‐methylated analogue comprises a full cyclohexylammonium cation, a full 18‐crown‐6, and a full ClO_4_^−^ anion (Figure 2a,b). The *trans*‐4‐methylcyclohexylammonium cation possesses a larger dipole moment than cyclohexylammonium (Figure S5), imparting higher polarity to the crystal. Moreover, its extended and more flexible geometry creates a relatively looser packing environment for the guest molecules and lowers the rotational energy barrier. This is reflected in the larger unit‐cell parameters along the projection direction of the cyclohexyl groups (*b* = 13.4530, *c *= 16.0336 Å for the methylated compound vs. *a* = 13.2932, *c* = 14.2024 Å for the non‐methylated one; Table S1), which facilitates the motion of host‐guest components and counterions.

Interaction‐region‐indicator (IRI) isosurface maps visually depict the chemical bonding and weak‐interaction regions in [(CH_3_‐C_6_H_10_‐NH_3_)(18‐crown‐6)][ClO_4_] (Figure 2c). The color scheme *sign*(λ_2_)*ρ* allows straightforward identification: blue regions indicate relatively strong attractive interactions, red regions denote repulsive interactions, and green regions correspond to very weak van der Waals (vdW) interactions [33, 34, 35]. As shown in Figure 2c and Figure S6, the isosurfaces around the ClO_4_^−^ anions interacting with the *trans*‐4‐methylcyclohexylammonium cation and the 18‐crown‐6 host change notably, with weakened C─H···O hydrogen bonds and expanded vdW interactions. These changes promote rotational disorder of the 18‐crown‐6 stator and ClO_4_^−^ anions at room temperature, accompanied by a low‐temperature phase transition. Between the cyclohexyl group and the 18‐crown‐6 macrocycle, the IRI maps display blue and green isosurfaces, confirming strong hydrogen‐bonding and weak vdW interactions, respectively, further supported by a denser hydrogen‐bond network (Figure S7). The steric effect of the methyl group is also enhanced. The denser and more extensive hydrogen‐bond network significantly increases the lattice energy, endowing the crystal with high thermal stability, which is consistent with the elevated Curie temperature observed in thermal analysis.

At 100 K (LTP), the 18‐crown‐6 molecules and one of the two ClO_4_^−^ anions in [(CH_3_‐C_6_H_10_‐NH_3_)(18‐crown‐6)][ClO_4_] become fully ordered, while the remaining ClO_4_^−^ anion remains rotationally disordered. The asymmetric unit now contains two *trans*‐4‐methylcyclohexylammonium cations, two 18‐crown‐6 molecules, and two ClO_4_^−^ anions, and the structure adopts a monoclinic space group *P*2_1_ (point group 2) (Figure 2d; Table S1). Despite the symmetry change, the overall stacking configuration resembles that of the ITP (Figure 2e,f). According to the Aizu rule, the transition between point groups *mm*2 and 2 corresponds to one of the 94 ferroelastic phase transitions (*mm*2*F*2) [36], indicating that methyl substitution introduces multiferroic behavior and enriches the physical properties.

**FIGURE 3:**
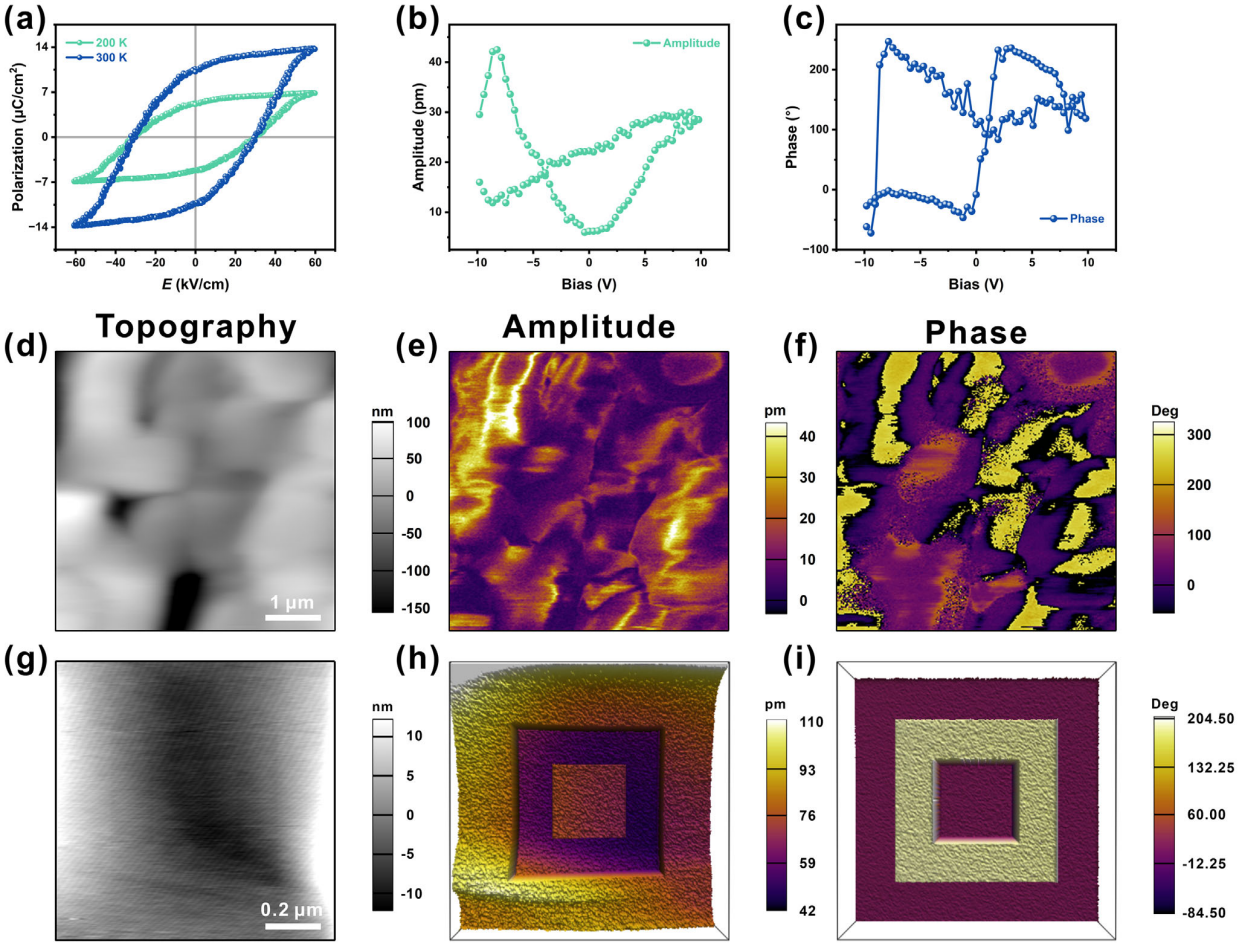
(a) Polarization‐electric field (*P*‐*E*) hysteresis loops of [(CH_3_‐C_6_H_10_‐NH_3_)(18‐crown‐6)][ClO_4_] crystals. (b,c) Amplitude‐voltage and phase‐voltage loops of crystal thin films. (d–f) Topographic, amplitude and phase images of [(CH_3_‐C_6_H_10_‐NH_3_)(18‐crown‐6)][ClO_4_] crystal thin films for 5 × 5 µm region. (g–i) Domain switching images of piezoelectric topographic, amplitude, and phase recorded within a 1 × 1 µm region under the tip bias of ±15 V.

To characterize the macroscopic ferroelectric polarization, regularly shaped single crystals were electroded along the *c*‐axis. Double‐wave method measurements yielded well‐saturated polarization‐electric field (*P‐E*) hysteresis loops (Figure 3a; Figure S8). The saturation polarizations (*P*_s_) are approximately 11.73 µC/cm^2^ at ITP and 13.81 µC/cm^2^ at LTP, in good agreement with values calculated from the crystal structures (Tables S2 and S3). These *P*_s_ values are the highest reported for supramolecular complex ferroelectrics, surpassing those of high‐temperature analogues such as [(NH_3_‐TEMPO)(18‐crown‐6)][ReO_4_] (∼6.2 µC/cm^2^) and [(C_6_H_11_‐NH_3_)(18‐crown‐6)][ClO_4_] (∼3.27 µC/cm^2^) [31, 32]. Ferroelectricity was further verified by piezoresponse force microscopy (PFM) on crystal thin films. When a conductive tip with an applied voltage scans the surface, the local piezoresponse (amplitude) and polarization orientation (phase) are recorded. The amplitude versus DC bias displays a characteristic butterfly loop, while the phase shows a clear hysteresis loop, confirming polarization switching (Figure 3b,c). Topography and PFM images (Figure 3d–f) reveal that the domain pattern is independent of surface morphology; the presence of 180° domains further attests to the ferroelectric nature. Polarization switching was directly demonstrated by writing domains with a PFM tip at ±15 V. As shown in Figure S3g–i, a uniform single‐domain region was first imaged as the initial state. After applying tip biases of opposite polarity, hollow‐square patterns with distinct phase contrast appeared, confirming stable and switchable ferroelectric polarization in [(CH_3_‐C_6_H_10_‐NH_3_)(18‐crown‐6)][ClO_4_].

**FIGURE 4:**
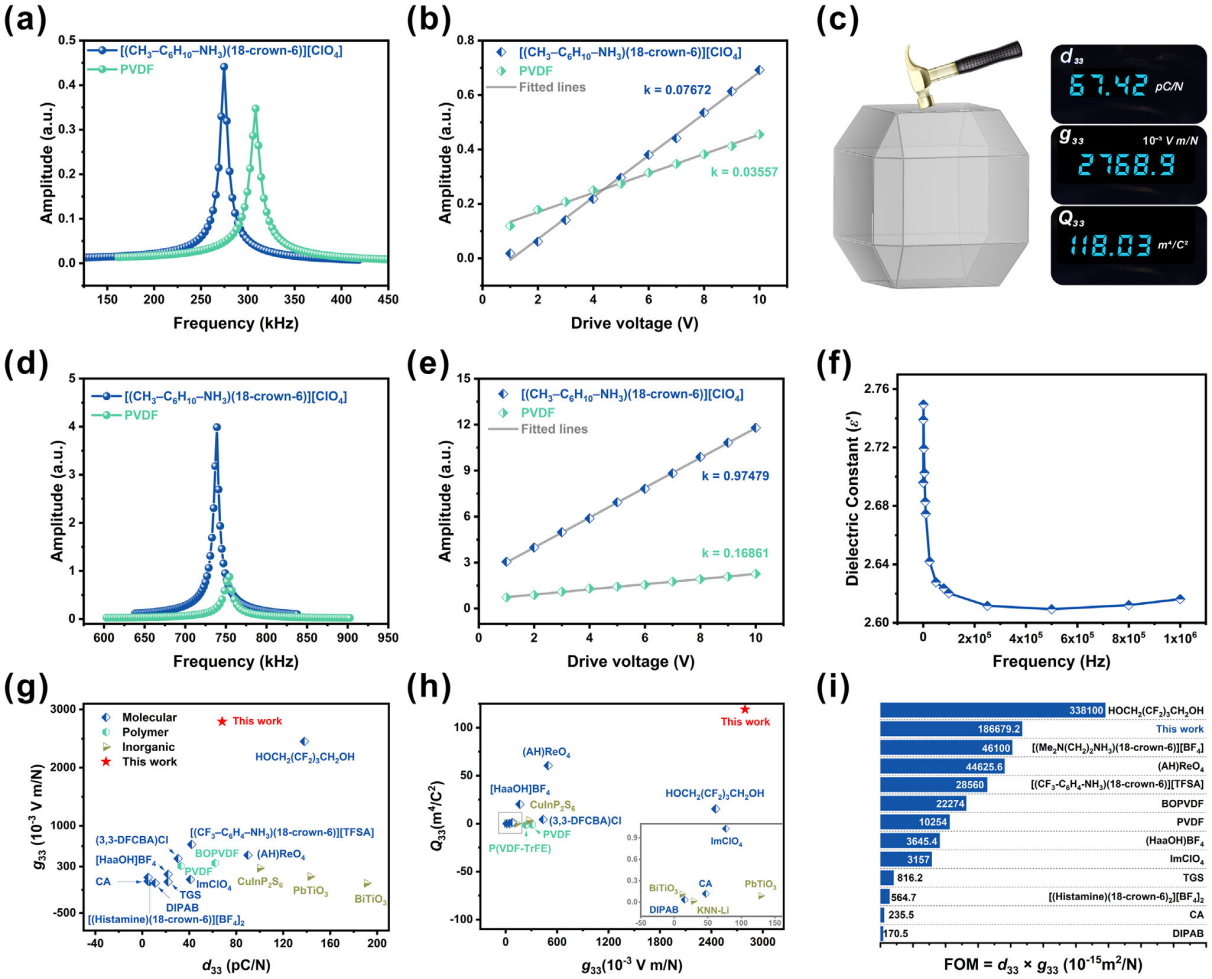
Comparison of (a) the vertical PFM resonance peaks and (b) the effective piezoelectric coefficient of [(CH_3_‐C_6_H_10_‐NH_3_)(18‐crown‐6)][ClO_4_] and PVDF films. (c) Diagram of the longitudinal piezoelectric coefficients of single crystals along the [001] direction using the quasi‐static method. Comparison of (d) the lateral PFM resonance peaks and (e) the effective piezoelectric coefficient. (f) Relative dielectric permittivity (ε′) as a function of frequency. (g,h) Comparison of piezoelectric coefficient (*d*_33_), piezoelectric voltage coefficient (*g*_33_) and electrostrictive coefficient (*Q*_33_). (i) FOM compared with the reported metal‐free molecular materials.

The piezoelectric performance of [(CH_3_‐C_6_H_10_‐NH_3_)(18‐crown‐6)][ClO_4_] was investigated at the microscale using piezoresponse force microscopy (PFM). Vertical and lateral PFM amplitudes were obtained by tracking the resonant surface displacements under a driving voltage; the amplitude parameter reflects the piezoresponse intensity. As shown in Figure 4a,d, the amplitude‐frequency response curves are well‐fitted by the damped harmonic oscillator model, and the piezoresponse varies linearly with drive voltage from 1 to 10 V, confirming intrinsic piezoelectric behavior. The slopes of the amplitude‐voltage plots represent effective piezoelectric coefficients (Figure 4b,e). In the vertical mode, the slope for [(CH_3_‐C_6_H_10_‐NH_3_)(18‐crown‐6)][ClO_4_] is approximately 2.16 times higher than that of poly(vinylidene fluoride) (PVDF, *d*_33_ = 33 pm/V), yielding an effective *d*_33_ of ∼71.18 pm/V. For the lateral mode, the slope is 5.78 times higher than that of PVDF (*d*_31_ = 21.4 pm/V), corresponding to an effective transverse piezoelectric coefficient *d*_31_ of ∼23.72 pm/V. To further evaluate the piezoelectric response, the quasi‐static method was employed to measure the longitudinal d_33_ along the [001] crystal axis. At room temperature, the obtained value is 67.42 ± 1.87 pC/N (Figure 4c; Figure S9 and Table S4), which represents the highest *d*_33_ reported for supramolecular complex ferroelectrics. This value surpasses those of [(*N*,*N*‐dimethylethylenediammonium)(18‐crown‐6)]BF_4_] (46.1 pC/N) [37], [(CF_3_−C_6_H_4_−NH_3_)(18‐crown‐6)][TFSA] (42 pC/N) [27] and [(NH_3_‐TEMPO)([18]crown‐6)](ClO_4_) (17 pC/N) [32] and also exceeds most typical metal‐free molecular ferroelectrics such as NDABCO‐NH_4_‐Br_3_ (63‐73.6  pC/N) [38], ImClO_4_ (41 pC/N) [39], MDABCO‐NH_4_‐I_3_ (∼14 pC/N, 14.4 pC/N) [40, 41], and triglycine sulfate (TGS, 22 pC/N) [20, 42].

In addition to a large *d*_33_, the *g*_33_ is also crucial for device applications. According to the relation *g*_33_ = *d*_33_/(ε′×ε_0_), where ε_0_ is the vacuum permittivity and ε′ is the relative permittivity along the polar direction, *g*_33_ can be directly evaluated. From the perspective of dielectric‐frequency behavior, interface polarization and all other polarization mechanisms contribute to the response in the low‐frequency region, resulting in dielectric responses accompanied by significant dielectric loss. Conversely, only rapid polarization mechanisms such as electron polarization and ion polarization can keep pace with the alternating electric field in the high‐frequency regime. Consequently, the dielectric permittivity approaches the material's intrinsic value, and dielectric loss is significantly reduced. To obtain an accurate ε′, large‐size crystals were measured along the [001] axis from 20 Hz to 1 MHz at room temperature. As shown in Figure 4f, ε′ is approximately 2.75 at 1 kHz, a value consistent with polycrystalline powder measurements (Figure 1b) and thin‐film capacitance data (Figure S10), confirming the reliability of the dielectric data. The resulting *g*_33_ is ∼2768.9 × 10^−3^ V m N^−^^1^, the highest value among crown‐ether‐based supramolecular ferroelectrics and one of the highest recorded for any metal‐free molecular ferroelectric. This figure exceeds those of HOCH_2_(CF_2_)_3_CH_2_OH (2450 × 10^−3^ V m/N) [24], [(CF_3_‐C_6_H_4_‐NH_3_)(18‐crown‐6)][TFSA] (680 × 10^−3^ V m/N) [27], (3,3‐DFCBA)Cl (437.2 × 10^−3^ V m/N) [43], (HaaOH)BF_4_ (165.7 × 10^−3^ V m/N) [44], ImClO_4_ (77 × 10^−3^ V m/N) [39], and DIPAB (15.5 × 10^−3^ V m/N) [45]. Moreover, it is notably larger than many metal‐based molecular ferroelectrics (e.g., TMCM‐MnCl_3_, 1681 × 10^−3^ V m/N; TMCM‐GaCl_4_ 1318 ×10^−3^ V m/N) [20, 46, 47, 48, 49], typical inorganic ferroelectrics (PbTiO_3_, 129 × 10^−3^ V m/N; CuInP_2_S_6_, 272 × 10^−3^ V m/N) [12, 50], and polymer ferroelectrics (PVDF, 310 × 10^−3^ V m/N; BOPVDF, 359 × 10^−3^ V m/N) [11, 51, 52]. As illustrated in Figure 4g, while classical ferroelectric ceramics possess large *d*_33_, their *g*_33_ values are typically low; conversely, ferroelectric polymers exhibit higher *g*_33_ but lower *d*_33_. Remarkably, molecular ferroelectrics such as [(CH_3_‐C_6_H_10_‐NH_3_)(18‐crown‐6)][ClO_4_] simultaneously offer both high *d*_33_ and high *g*_33_, making them highly attractive for piezoelectric applications.

The electrostrictive coefficient *Q*_33_, another key parameter for electromechanical coupling, was derived from the intrinsic piezoelectric relation *d*_33_ = 2*P_s_* ε_33_ *Q*_33_. The calculated *Q*_33_ for [(CH_3_‐C_6_H_10_‐NH_3_)(18‐crown‐6)][ClO_4_] is ∼ 118.03 m^4^/C^2^ (Table S6), among the highest for metal‐free molecular ferroelectrics, higher than some metal‐free molecular ferroelectrics (TMCM‐CdBrCl_2_, 100.2 m^4^/C^2^ [18], [C_6_H_5_N(CH_3_)_3_]CdBr_2_Cl_0.75_I_0.25_, 64.2 m^4^/C^2^ [16], (TMFM)_0.26_(TMCM)_0.74_CdCl_3_, 63.37 m^4^/C^2^ [19]) and nearly two orders of magnitude greater than those of polymer P(VDF‐TrFE) (‐1.5  m^4^/C^2^) and inorganic CuInP_2_S_6_ (3.13 m^4^/C^2^) (Figure 4h) [53, 54] Furthermore, the figure of merit (FOM = *d*_33_ ×*g*_33_) reaches 1.866792 × 10^−10^ m^2^/N, which ranks it among the highest for metal‐free molecular ferroelectrics and is one to two orders of magnitude larger than those of PVDF‐ and PZT‐based harvesters (Table S6; Figure 4i). These outstanding metrics underscore the material's potential for high‐performance piezoelectric energy harvesting and sensing.

**FIGURE 5:**
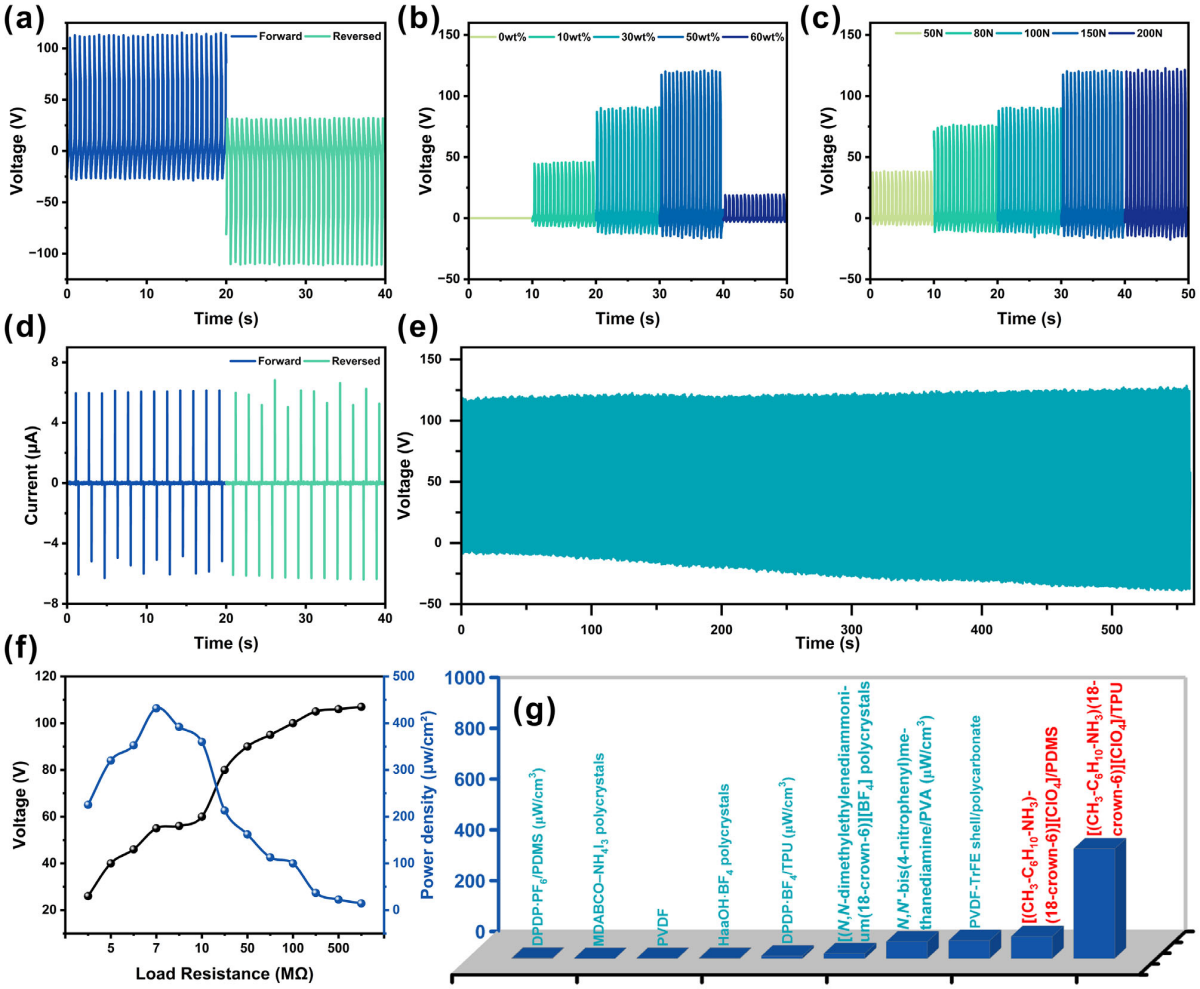
Piezoelectric energy harvesting performance of [(CH_3_‐C_6_H_10_‐NH_3_)(18‐crown‐6)][ClO_4_]/TPU composite films. (a) and (d) The polarization switching output voltage (*V*_oc_) and short‐circuit current (*I*_sc_) of 50% composite film with 150 N (1.7 Hz). (b) The piezoelectric energy harvester outputs *V*_oc_ signals of the piezoelectric energy harvesters with different contents. (c) *V*_oc_ of piezoelectric energy harvester output for 50% composite films under applying force from 50 to 200 N. (e) The long‐term output performance was tested for 560 s. (f) The resistance‐dependent voltages and power densities under 150 N. (g) Comparison of the power density with reported metal‐free and mechanical harvesting devices.

To assess the energy‐harvesting capability of [(CH_3_‐C_6_H_10_‐NH_3_)(18‐crown‐6)][ClO_4_] for piezoelectric applications, flexible porous composite devices (1 × 1  cm^2^, 0.5 cm thick) were fabricated by embedding the piezoelectric crystals into a thermoplastic polyurethane (TPU) matrix. Field‐emission scanning electron microscopy (FE‐SEM) images of the cross‐section reveal a loose, porous TPU network with uniformly dispersed ferroelectric crystals in the 50 wt.% composite (Figure S11a). Energy‐dispersive X‐ray spectroscopy (EDS) mapping confirms the homogeneous distribution of C, O, N, and Cl elements throughout the framework (Figures S11b–e), verifying the successful integration of [(CH_3_‐C_6_H_10_‐NH_3_)(18‐crown‐6)][ClO_4_] within the TPU skeleton without alteration of its chemical composition. Dielectric characterization indicates that the piezoelectric output of the composites arises not only from the intrinsic piezoelectric effect but also from interfacial polarization contributions at low frequencies and elevated temperatures (Figures S12 and S13). Leveraging the high porosity and flexibility of TPU, a series of sandwich‐structured devices with varying [(CH_3_‐C_6_H_10_‐NH_3_)(18‐crown‐6)][ClO_4_] loadings (0, 10, 30, 50, and 60 wt.%) were prepared, using copper foils as top and bottom electrodes. The piezoelectric outputs were measured under a compressive force of 150 N at 1.7 Hz (Figure S14). Polarity‐switching tests demonstrate that the open‐circuit voltage (*V*_oc_) and short‐circuit current (*I*_sc_) originate from the intrinsic piezoelectric response of the crystals: reversing the electrical connections inverts the output signals, and the magnitudes remain nearly symmetric between forward and reversed configurations (Figure 5a,d). This confirms the stability and reliability of the composite devices. The output performance depends strongly on the piezoelectric loading (Figure 5b; FigureS15). For instance, the device with 10 wt.% content delivers *V*_oc_  ≈ 46 V and *I*_sc_  ≈ 2 µA. As the loading increases from 10 to 50 wt.%, both *V*_oc_ and *I*_sc_ rise linearly, reflecting enhanced piezoelectric activity under the same mechanical stimulus. The optimal performance is achieved at 50 wt.%, yielding a maximum *V*_oc_ of 120 V and *I*_sc_ of 6.1 µA. Further increasing the loading to 60 wt.% reduces the output due to inhomogeneous dispersion and localized aggregation of the crystals, which impedes efficient stress transfer.

To further evaluate the piezoelectric performance under varying mechanical stimuli, the 50 wt.% [(CH_3_‐C_6_H_10_‐NH_3_)(18‐crown‐6)][ClO_4_]/TPU porous composite device was subjected to periodic compressive forces ranging from 50 N to 200 N at 1.7 Hz. As shown in Figure 5c and Figure S15, both the *V*_oc_ and *I*_sc_ increase linearly with the applied force up to 150 N. Under a moderate force of 50 N, representative of common environmental vibrations, the device delivers a considerable output of *V*_oc_ ≈ 39 V and *I*_sc_ ≈ 2.1 µA. Remarkably, after 8 months of ambient exposure, the device retained nearly its original performance (*V*_oc_ ≈ 35 V, *I*_sc_ ≈ 2.1 µA) over a 1200 s test at 1.63 Hz (Figure S16 and S17), demonstrating exceptional long‐term stability. At the optimal compressive force of 150 N, the device reaches its maximum output: *V*_oc_ ≈ 120 V and *I*_sc_ ≈ 6.1 µA. This linear force‐response relationship, together with the high voltage tolerance, underscores the material's suitability for high‐energy‐density applications. Compared with other reported flexible energy harvesters, the *V*_oc_ of our porous composite is among the highest, rivaling outputs from PVDF‐TrFE‐shell/polycarbonate (126 V), gradient‐porous/PZT/PDMS (152 V), and TMCM‐CdCl_3_/TPU (103 V) systems (Table S7) [23, 55, 56]. More importantly, it represents the highest *V*_oc_ reported for any metal‐free molecular piezoelectric or fluorinated imine‐based 2D covalent organic framework ferroelectric [57]. The device also maintains stable operation for over 560 s under 150 N compression without significant signal decay (Figure 5e), and after 8 months, a *V*_oc_ of 119 V persists for 1200 s with no decline (Figure S18), confirming outstanding mechanical and environmental durability.

For practical energy‐harvesting assessment, the output was measured across external load resistances (*R*) from 3 MΩ to 800 MΩ. The voltage rises with increasing *R*, and the power density (*P* = *V* ^2^/(*R* × *S*)) reaches 16 µW/cm^2^ at 9 MΩ under 50 N (Figure S16c). At 150 N, the maximum power density attains 432.1 µW/cm^2^ at 7 MΩ (Figure 5f; Table S7), which is the highest value among all metal‐free molecular ferroelectrics (Figure 5g). This performance surpasses that of [(*N*, *N*‐dimethylethylenediammonium)(18‐crown‐6)][BF_4_] polycrystals (19.6 µW/cm^2^) [37], (HaaOH)BF_4_ (1.2 µW/cm^2^) [44], DPDP·BF_4_/TPU (10.16 µW/cm^2^) [58], PVDF‐TrFE shell/PC composite (71 µW/cm^2^) [55], and many inorganic‐ and metal‐based molecular ferroelectric composites [56, 59, 60, 61], except for TMCM‐CdCl_3_/TPU (636.9 µW/cm^2^) [22, 23, 29, 30, 61]. A PDMS‐based composite film also exhibits notable outputs (*V*_oc_ ≈ 6 V, *I*_sc_ ≈ 31 µA, power density ≈ 86.49 µW/cm^2^; Figure S19 and S20), confirming the versatility and superior piezoelectricity of [(CH_3_‐C_6_H_10_‐NH_3_)(18‐crown‐6)][ClO_4_].

**FIGURE 6:**
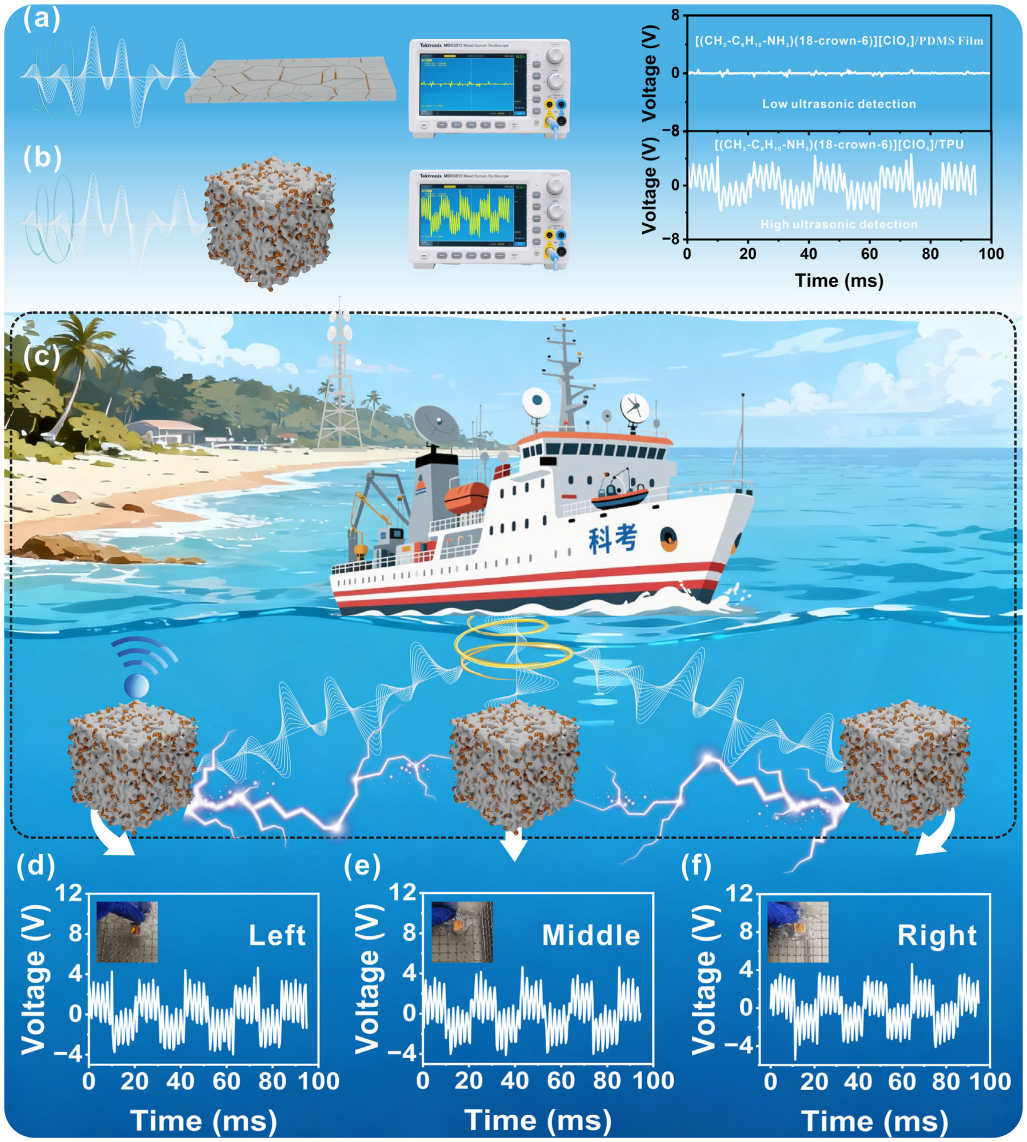
The application of [(CH_3_‐C_6_H_10_‐NH_3_)(18‐crown‐6)][ClO_4_] composite devices for underwater ultrasonic wave detection. (a) and (b) The ultrasonic detection models and voltage diagrams of 1% [(CH_3_‐C_6_H_10_‐NH_3_)(18‐crown‐6)][ClO_4_]/PDMS composite film and 50% [(CH_3_‐C_6_H_10_‐NH_3_)(18‐crown‐6)][ClO_4_]/TPU porous composite, respectively. (c) Schematic diagram of the self‐powered ultrasonic sensor network detecting waves across different underwater regions. (d–f) The voltage outputs of 50% [(CH_3_‐C_6_H_10_‐NH_3_)(18‐crown‐6)][ClO_4_]/TPU porous composites are recorded in the left, middle, and right regions of the water tank, respectively.

Owing to their excellent mechanical compatibility, efficient stress transfer, and high power density, porous piezoelectric composites hold great promise for advanced applications such as underwater ultrasonic detection. The inherent acoustic‐energy‐absorption capability of porous materials (Figure 6a,b) allows them to be integrated into a multi‐unit sensor array for aquatic environments (Figure 6c). In a prototype setup, porous composite units placed at different positions (left, middle, right) in a water tank generated stable voltage signals of approximately 4 V upon ultrasonic excitation, with minimal variation across locations (Figure 6d–f). These signals show no attenuation after 8 months and remain constant over 500 ms (Figure S21). By processing the electrical signals from all units, comprehensive ultrasonic monitoring of the entire water volume can be achieved, demonstrating the practical potential of these porous composites for robust, high‐sensitivity underwater detection.

## 3 Conclusion

In summary, this work reports the metal‐free molecular ferroelectric [(CH_3_‐C_6_H_10_‐NH_3_)(18‐crown‐6)][ClO_4_] by a methylation strategy to enhance piezoelectricity. The compound demonstrates outstanding piezoelectric performance, with a large *d*_33_ of 67.42 ± 1.87 pC/N and a remarkably high *g*_33_ of ∼ 2768.9 × 10^−3^ V m/N, yielding a FOM of 1.87 × 10^−10^ m^2^/N that surpasses nearly all known molecular ferroelectrics. When integrated into a flexible porous composite with thermoplastic polyurethane (TPU), the material delivers a high open‐circuit voltage of 120 V and a record power density of 432.1 µW/cm^2^ among metal‐free molecular piezoelectrics. These properties, combined with excellent long‐term stability, highlight its strong potential for applications in flexible electronics, wearable sensors, and biomechanical energy harvesting. Future studies should focus on the temperature‐dependent stability of its piezoelectric and dielectric responses, as well as the environmental endurance of composite devices, to advance the material toward practical use in demanding environments.

## Funding

The authors acknowledge funding support from the National Natural Science Foundation of China (Grant Nos. 12364012 and 22001102) and the Bagui Scholars Programme of Guangxi Province.

## Conflicts of Interest

The authors declare no conflicts of interest.

## Supporting information




**Supporting file**: advs74606‐sup‐0001‐SuppMat.docx

## Data Availability

The data that support the findings of this study are available from the corresponding author upon reasonable request.
