# Supplementary material for: A Metal‐Free Molecular Ferroelectric With Large Piezoelectricity and Its Porous Composite for Superior Output Power Density
Source: Adv Sci (Weinh). 2026 Mar 2;13(27):e24032. doi: 10.1002/advs.202524032 (PMC13170225; doi:10.1002/advs.202524032)
Supplement: Supplementary file 1 — Supporting file: advs74606‐sup‐0001‐SuppMat.docx [file ADVS-13-e24032-s001.docx]

**Supporting information for**

**A Metal-free Molecular Ferroelectric with Large Piezoelectricity and Its Porous Composite for Superior Output Power Density**

Nan Chen,^[a],†^ Wen-Juan Wei,^*[a],[b],†^ Le Ye,^[c],†^ Ling-Yu Wan,^*[a]^ and Yen Wei^*[b]^

^[a]^ *Center on Nanoenergy Research, School of Physical Science and Technology, Guangxi University, Nanning 530004, China*

^[b]^ *Department of Chemistry and the Tsinghua Center for Frontier Polymer Research, Tsinghua University, Beijing 100084, China*

^[c]^ *Department of Chemistry, Southern University of Science and Technology, Shenzhen, Guangdong, 518055, China*

^†^ These authors contributed equally to this work.

Corresponding Author: [wwenjuan20@163.com](mailto:wwenjuan20@163.com) (W.W.); [lywan2017@gxu.edu.cn](mailto:lywan2017@gxu.edu.cn) (L.W.); [weiyen@tsinghua.edu.cn](mailto:weiyen@tsinghua.edu.cn) (Y.W.).

**Materials and Methods**

**Material synthesis.**

All chemicals and solvents were of reagent grade and used as received. The [(CH_3_-C_6_H_10_-NH_3_)(18-crown-6)][ClO_4_] and [(C_6_H_11_-NH_3_)(18-crown-6)][ClO_4_] single crystals are synthesized by dissolving the corresponding chemical ratio of reagents in a methanol solution. After several days of slow evaporation, resulting in the formation of colorless block single crystals.

**XRD measurements.**

Single crystal X-ray diffractions were carried out with graphite monochromated Mo radiation (*λ* = 0.71073 Å) on Rigaku Oxford Diffraction diffractometer. Data sets were collected by using *CrysAlis^Pro^* software. The program Olex2-1.2 was employed as an interface to invoke program SHELXS97 and SHELXL97 executables. The crystal structures were solved by direct methods with SHELXTL and refined by full-matrix least squares on *F^2^* with anisotropic atomic displacement parameters for all non-hydrogen atoms using SHELXL97. All H atoms were located from molecular geometric calculations and refined with isotropic temperature parameters. The crystal parameters and structure refinement are summarized in Table S1. The crystallographic structures determined at temperatures far from their respective phase transition have been deposited in the CIF format in the Cambridge Crystallographic Database Centre as supplementary material, CCDC 2491005-2491007. Powder X-ray diffraction (PXRD) data were measured using a Rigaku D/MAX 2000 PC *X*-ray diffraction system with Cu *Kα* radiation in the 2*θ* range of 5^°^-60^°^ with a step size of 0.02^°^.

**TGA, DSC and Dielectric measurements.**

Thermogravimetric analysis (TGA) was performed using an SDT-Q600 thermal analyzer (TA instrument) at a heating rate of 10 K·min^-1^ under an air atmosphere in alumina crucibles. Differential scanning calorimetry (DSC) measurements were recorded on a TA Q2000 DSC instrument by heating and cooling crystalline samples with a rate of 10 K·min^-1^ under in aluminum crucibles at nitrogen atmosphere. Dielectric measurements were implemented with powder samples in the form of tablet which used as electrodes by pasting silver conduction on its both surfaces compactly. The dielectric constants of the compounds were confirmed with an Agilent or a Model TH2828A impedance analyzer over the frequency range from 0.8 kHz to 1 MHz. To accurately determine the relative permittivity (${}_{r}$), the capacitance of a parallel-plate capacitor of sample pellets was measured under vacuum condition and the derived ${}_{r}$ was ca. 2.74 according to the equation of ${}_{r}$= (*C*×*d*)/(${}_{0}$×*S*) (${}_{0}$ is the vacuum permittivity, *C* is capacitance, *d* is the thickness and *S* is the area of the sample), which is relatively close to the measured dielectric constant value (ca. 2.75).

**SHG and Polarization–voltage measurements.**

Second harmonic generation (SHG) measurements were carried out by using an integrated instrument, which ensured a low divergence (pulsed Nd:YAG at a wavelength of 1064 nm, 10 Hz repetition rate, 1.6 MW peak power, 5 ns pulse duration) for the unexpanded laser (OPOTEK, 355 II). SHG signals were measured at room temperature and compared with the standard potassium dihydrogen phosphate (KDP). Temperature-dependent SHG experiments were performed using powder samples with a temperature range of 152-452 K. A Radiant Precision Premier II analyzer was used in the polarization−electric field (*P*-*E*) hysteresis loop measurements which can be extracted from reversal current curve recorded by double-wave method. Typically, the block single crystals are coated with conduction silver glue perpendicular to the crystal axis as the electrodes (Figure S8).

**PFM and SEM characterization.**

The PFM measurements were carried out on a commercial piezoresponse force microscope (Cypher, Asylum Research). Conductive Pt/Ir-coated silicon probes (EFM-20, Nanoworld) with an AC drive voltage were used for domain imaging and polarization switching studies, which the nominal spring constant and resonance frequency are ~2.8 nN/nm and ~75 kHz, respectively. As an effective method for studying piezoelectric responses at the nanoscale, PFM estimates the piezoelectric response by measuring the vibration amplitude of a cantilever beam under a unit driving voltage. Specifically, the maximum amplitude is divided by the driving voltage to calculate the microscopic piezoelectric response. The morphology of the porous composite piezoelectric materials was observed by field emission scanning electron microscope (FE-SEM, America). Correspondingly, the energy-dispersive spectroscopy (EDS) mapping were observed.

$\boldsymbol{d}_{\boldsymbol{33}}$ **measurement.**

The macroscopic piezoelectric coefficient ($d_{33}$) was measured by a commercial piezometer (ZJ-6BN) using quasi-static method. All tests were conducted in a temperature- and humidity-controlled chamber. Prior to each test, the system was calibrated and zeroed using a calibration sample, followed by testing with a standard sample (~ 289 pC/N), with errors falling within the instrument's nominal range (<±2%). The sample crystal was placed in between two flat metal plates by applying a small oscillating force (3 N) along the normal direction under alternating force frequency (110 Hz) and amplitude (0.25 N).

**Preparation of thin-film, [(CH_3_-C_6_H_10_-NH_3_)(18-crown-6)][ClO_4_]/TPU dense composites and [(CH_3_-C_6_H_10_-NH_3_)(18-crown-6)][ClO_4_]/PDMS composite films.**

The thin films were prepared through the drop-casting method. The precursor solution of [(CH_3_-C_6_H_10_-NH_3_)(18-crown-6)][ClO_4_] was prepared by dissolving 0.118 g of the crystallized samples in 0.5 mL of the methanol solution. Then, 20 μL of the precursor solution was spread on a clean ITO-glass substrate (1.5 × 1.5 cm), and the high-quality thin films were obtained by slowly evaporated on a hot plate of 323 K for 1 h. Composites of [(CH_3_-C_6_H_10_-NH_3_)(18-crown-6)][ClO_4_] and thermoplastic polyurethane (TPU) were prepared via the non-solvent-induced phase separation (NIPS) method. First, TPU particles were continuously stirred in 1,4-dioxane at 60°C until a uniform transparent solution formed. Next, a predetermined mass fraction of [(CH_3_-C_6_H_10_-NH_3_)(18-crown-6)][ClO_4_] powder was added to the solution at mass fractions of 0 wt%, 10 wt%, 30 wt%, 50 wt%, and 60 wt%, respectively, and stirred until uniformly dispersed. The mixture was placed in a fume hood for solvent evaporation. When the solution thickened to a viscous state (approximately 50% solvent volume evaporated), excess room-temperature ethanol was added to induce instantaneous liquid-liquid phase separation, forming a white flocculent precipitate. The precipitate was filtered, collected, and compacted into a 1.6 cm × 1.6 cm × 1.6 cm silicone mold. The sample was first left at room temperature overnight for natural solvent evaporation, then placed in a vacuum oven for vacuum drying at room temperature for 3 hours to completely remove residual solvent. During drying, the sample shrinks significantly to 1.0 cm due to solvent evaporation, forming a uniform porous structure internally. This yields a highly elastic porous rectangular prism composite material measuring 1 cm × 1 cm × 0.5 cm. The [(CH_3_-C_6_H_10_-NH_3_)(18-crown-6)][ClO_4_]/PDMS composite films were prepared by adding uniformly ground [(CH_3_-C_6_H_10_-NH_3_)(18-crown-6)][ClO_4_] powder at mass fractions of 0.1%, 0.5%, 1%, 1.5%, and 5% to 2.5 g of PDMS prepolymer. Subsequently, 15 mL of dichloromethane (CH₂Cl₂) was added and stirred for 4 hours to form a homogeneous solution. Upon near-complete evaporation of CH₂Cl₂, 10 wt% (250 mg) of curing agent was added under stirring for pre-curing. The mixture was degassed in a vacuum oven to remove residual solvents and bubbles, then coated onto substrates to form uniform films.

**Fabrication of [(CH_3_-C_6_H_10_-NH_3_)(18-crown-6)][ClO_4_]/TPU and [(CH_3_-C_6_H_10_-NH_3_)(18-crown-6)][ClO_4_]/ PDMS devices.**

Firstly, conductive silver paste was applied to both sides of the [(CH_3_-C_6_H_10_-NH_3_)(18-crown-6)][ClO_4_]/TPU porous composites and [(CH_3_-C_6_H_10_-NH_3_)(18-crown-6)][ClO_4_]/ PDMS composite films, and copper foil was also adhered to it to fabricate a sandwich-type laminated structure device. Secondly, in order to improve the contact between the copper foil and the film, a press machine was used to compress the [(CH_3_-C_6_H_10_-NH_3_)(18-crown-6)][ClO_4_]/TPU and [(CH_3_-C_6_H_10_-NH_3_)(18-crown-6)][ClO_4_]/ PDMS devices to eliminate air gaps. Finally, the piezoelectric energy harvesting test was conducted directly at room temperature without polarization treatment.

**Ultrasound detection of [(CH_3_-C_6_H_10_-NH_3_)(18-crown-6)][ClO_4_] composite devices**

The experiments of the 50% [(CH_3_-C_6_H_10_-NH_3_)(18-crown-6)][ClO_4_]/TPU dense composite devices and 1% [(CH_3_-C_6_H_10_-NH_3_)(18-crown-6)][ClO_4_]/PDMS composite film devices were conducted using a numerically controlled ultrasonic cleaner (Model KQ-800DE, Shumei) with an inner tank dimension of 500 × 300 × 200 mm. Deionized water was filled to a height of 100 mm in the tank. The ultrasonic source, operating at a frequency of 40 kHz, was fixed at the center of the tank bottom. Both sides of each test sample were attached with copper foil as electrodes and connected with lead wires. The samples were sequentially placed at three predetermined positions inside the tank—left, middle, and right—with a spacing of 20 cm between them. The sample surface was oriented perpendicular to the direction of ultrasonic wave propagation, and the sample was immersed 50 mm below the water surface. The open-circuit voltage signals across the two ends of the sample were directly acquired using a digital oscilloscope. For each measurement, a stable 100 ms time-domain signal was recorded, and each position was tested three times. During signal processing, the peak-to-peak value of the voltage waveform within this 100 ms window was taken as the output amplitude for that measurement.

**Density functional theory (DFT) calculations.**

All first-principles calculations were performed using density functional theory (DFT). Molecular systems employed Gaussian 09 with the B3LYP hybrid functional, def2-TZVP basis set, and Grimme’s D3 dispersion correction (GD3BJ). The electrostatic potential involved in the analyses was evaluated by Multiwfn based on the highly effective algorithm proposed in Ref ^[^[^1^](#_ENREF_1)^]^. The rotation energies were calculated by the periodic DFT method using the Dmol3 module.

**The measurement of the energy conversion efficiency (η).**

In this experiment, a linear motor was used to apply pressure to the PENG sample. The input mechanical energy was calculated based on the force–displacement mode, which better reflects the actual pressure‑loading scenario. Essentially, this approach quantifies the input energy through mechanical work, aligning with the kinetic‑energy calculation logic adopted in references ^[^[^2^](#_ENREF_2)^]^. Both follow the same efficiency definition of “output electrical energy / input mechanical energy,” and the core calculation rule remains unchanged.

1. Energy conversion efficiency under 150N

Work input per cycle:$W_{Single Cycle}=F\times x=150\times3.071\times{10}^{-3}\approx\frac{0.4607J}{Cycle}$

Total input mechanical energy in 20 seconds:$E_{Mechanical}=W_{Single Cycle}\times f\times t=0.4607\times1.7\times20\approx15.66J$

Output power:$P_{Output}=\frac{V^{2}}{R}=\frac{{55}^{2}}{7\times{10}^{6}}\approx4.32\times{10}^{-4}W$

Total output electrical energy in 20 seconds:$E_{Electrical}=P_{Output}\times t=4.32\times{10}^{-4}\times20\approx8.64\times{10}^{-3}J$

Energy Conversion Efficiency:$\eta=\frac{E_{Electrical}}{E_{Mechanical}}\times100\%=\frac{8.64\times{10}^{-3}}{15.66}\times100\%\approx0.055\%$

1. Energy conversion efficiency under 50N

Work input per cycle:$W_{Single Cycle}=F\times x=50\times1.726\times{10}^{-3}\approx\frac{0.0858J}{Cycle}$

Total input mechanical energy in 20 seconds:$E_{Mechanical}=W_{Single Cycle}\times f\times t=0.0858\times1.63\times20\approx2.798J$

Output power:$P_{Output}=\frac{V^{2}}{R}=\frac{{12}^{2}}{9\times{10}^{6}}\approx1.6\times{10}^{-5}W$

Total output electrical energy in 20 seconds:$E_{Electrical}=P_{Output}\times t=1.6\times{10}^{-5}\times20\approx3.2\times{10}^{-4}J$

Energy Conversion Efficiency:$\eta=\frac{E_{Electrical}}{E_{Mechanical}}\times100\%=\frac{3.2\times{10}^{-4}}{2.798}\times100\%\approx0.019\%$


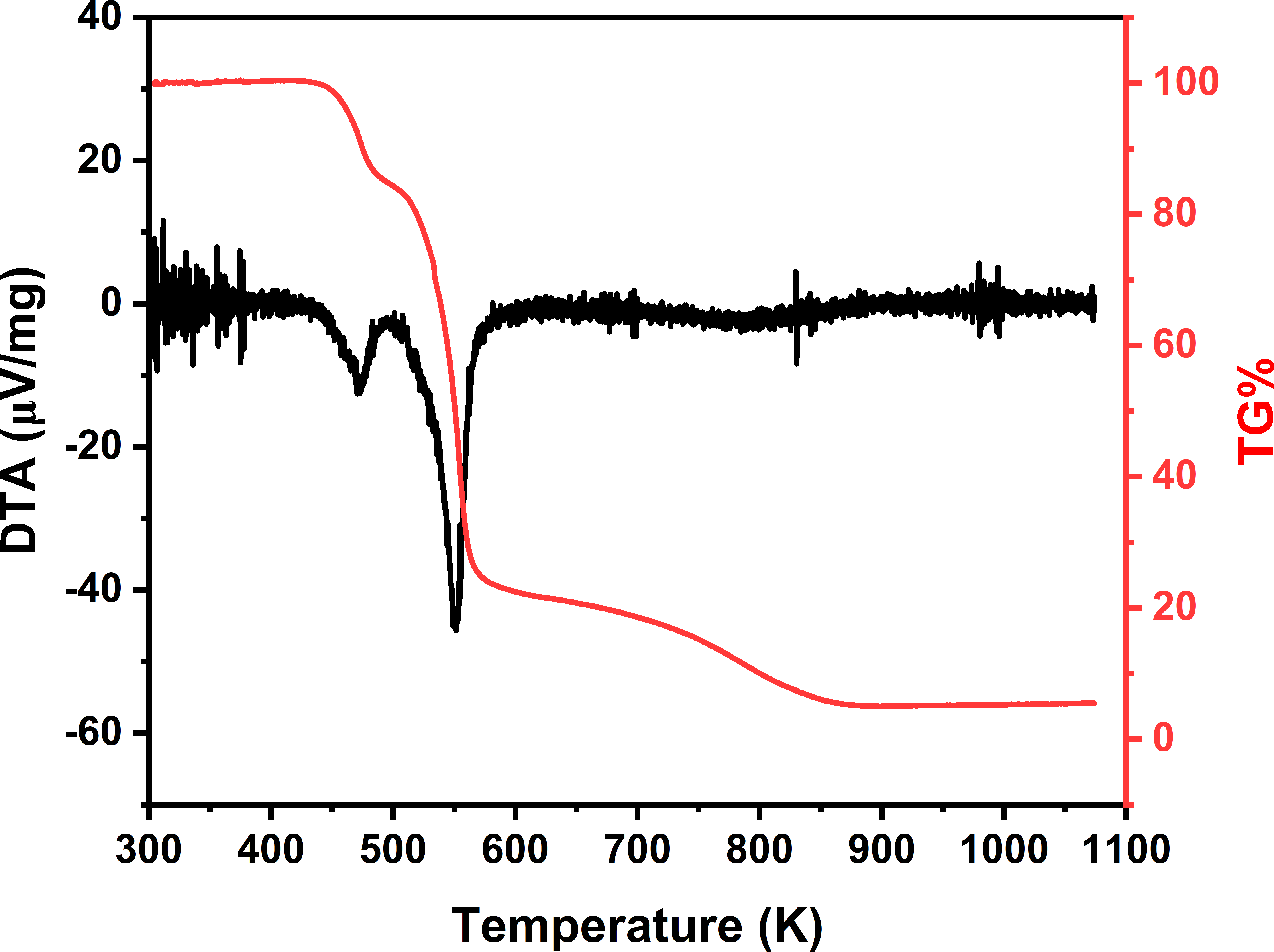


Figure S1. TG and DTA curve of [(CH_3_-C_6_H_10_-NH_3_)(18-crown-6)][ClO_4_].


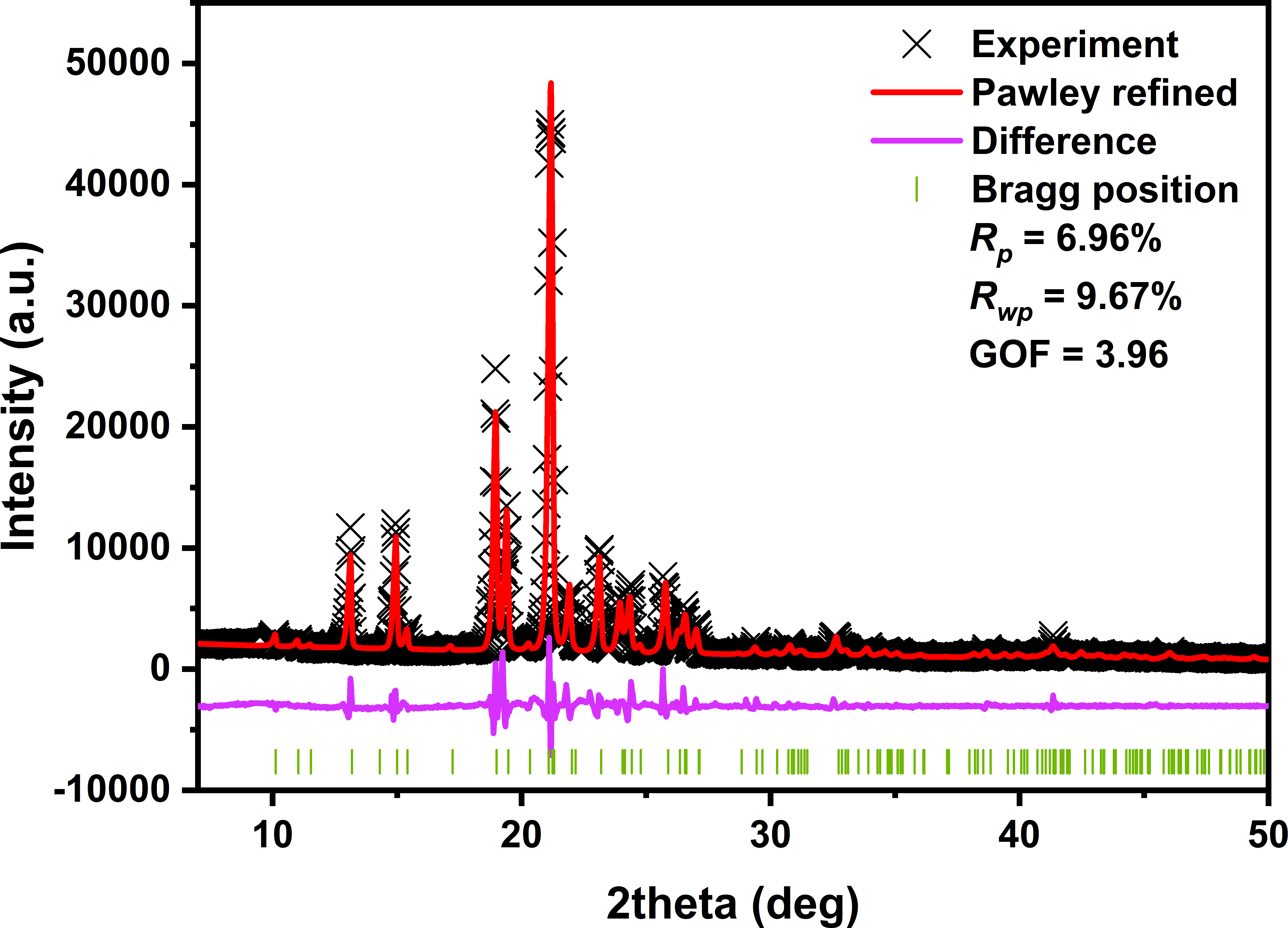


Figure S2. Powder XRD patterns of [(CH_3_-C_6_H_10_-NH_3_)(18-crown-6)][ClO_4_] refined by the Pawley method at room temperature. The black symbol, red and lilac continuous lines, pale green symbol are the experimental, calculated, difference, and bragg position profiles, respectively. *R_p_* = 6.96%, *R_wp_* = 9.67%, and GOF = 3.96.

*
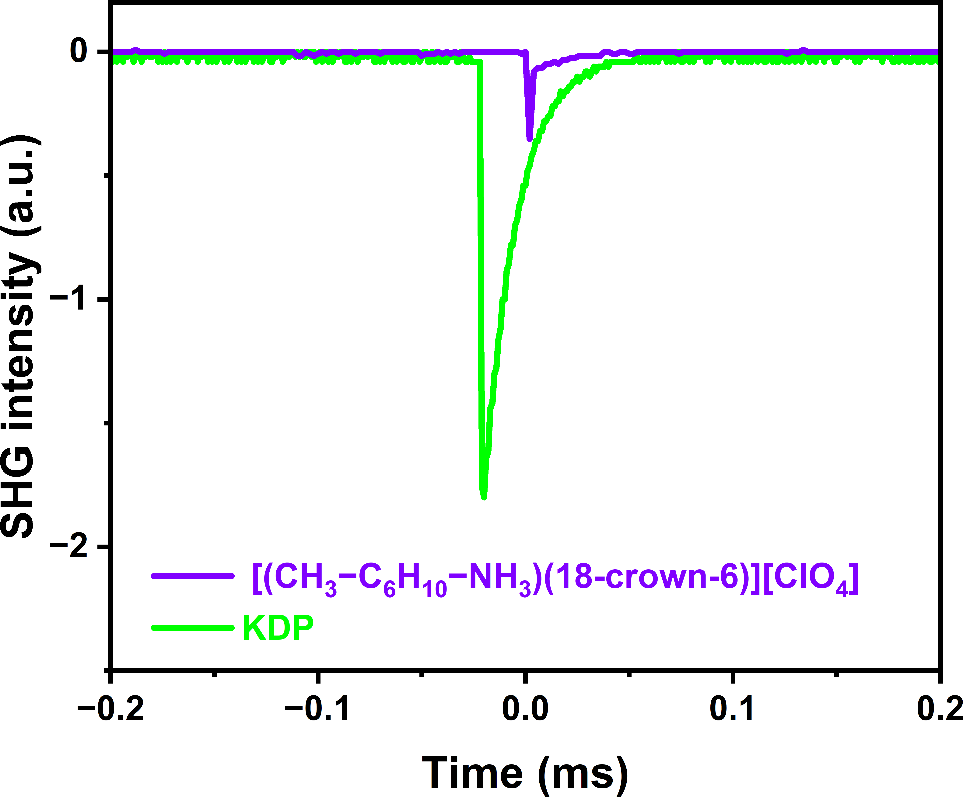
*

Figure S3. SHG signals for the synthesized crystals and KDP at room temperature.


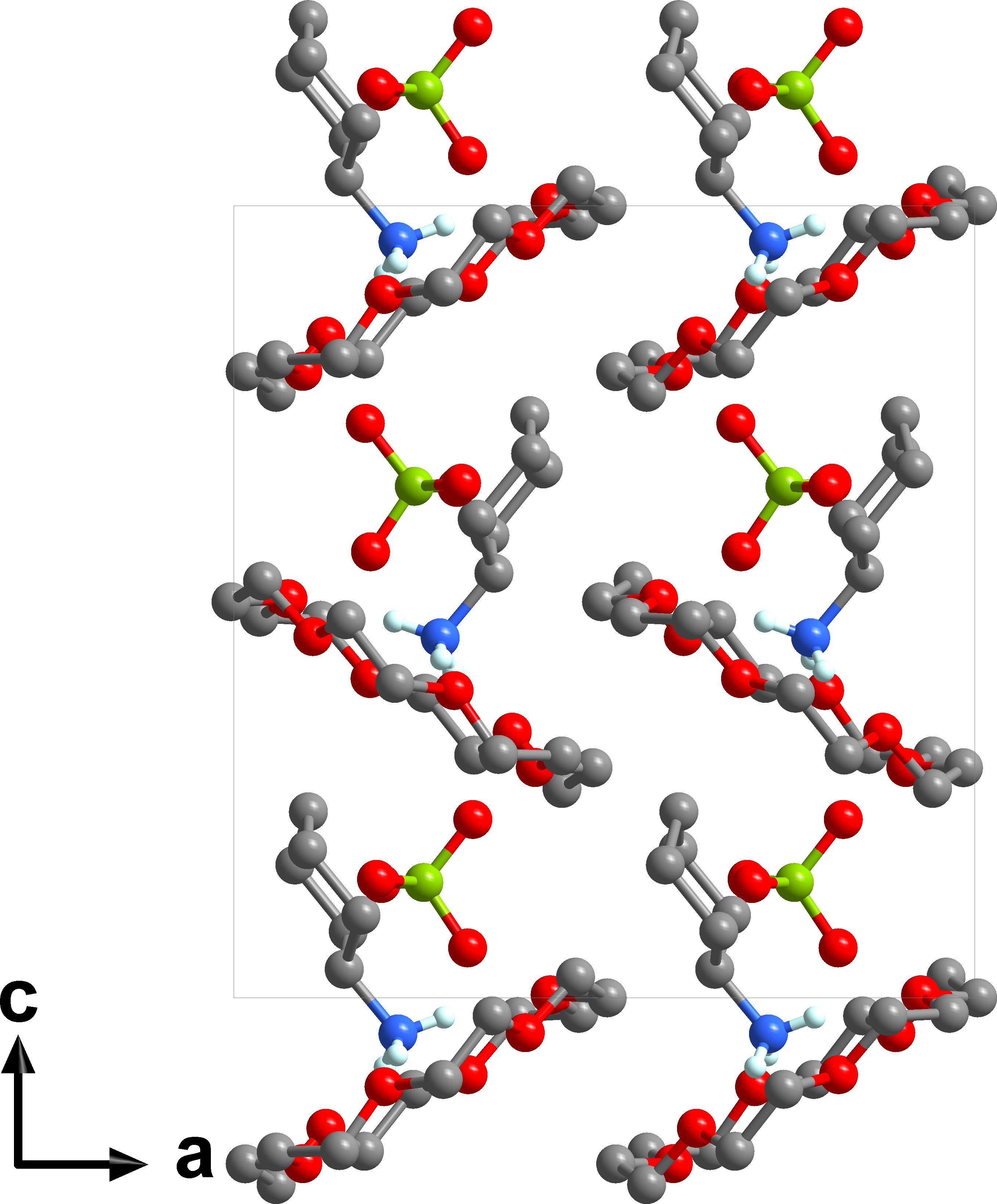


Figure S4. The packing view of structures of [(C_6_H_11_-NH_3_)(18-crown-6)][ClO_4_] at room temperature. Parts of hydrogen atoms were omitted for clarity.


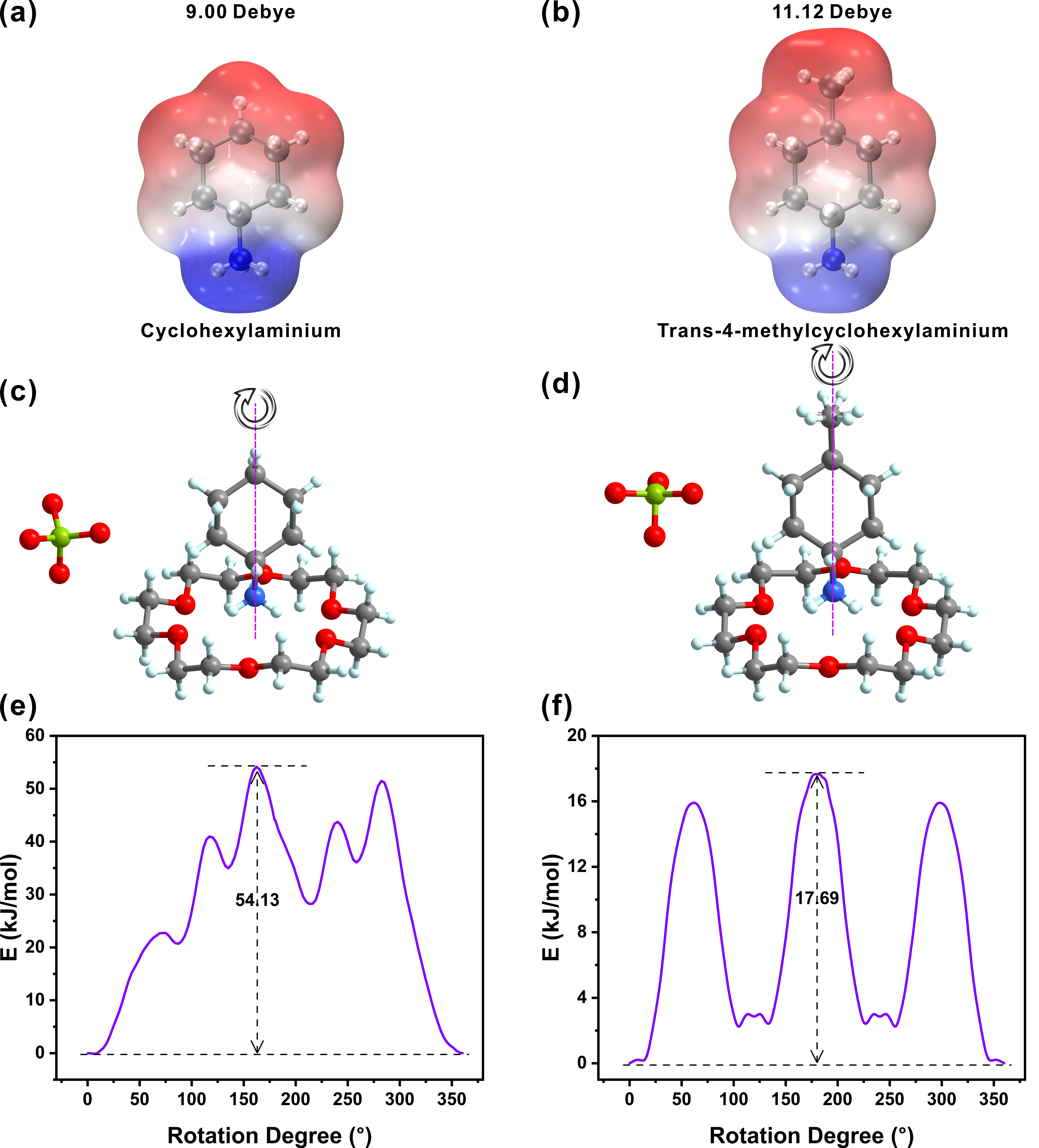


Figure S5. (a) and (b) Calculated dipole moments and electrostatic potentials of cyclohexylaminium and methylated cyclohexylaminium based on charge distribution analysis. (c) and (e) Schematic diagram of rotation and calculated rotational energy barrier diagram for the cyclohexylamine molecule in the structure [(C_6_H_11_-NH_3_)(18-crown-6)][ClO_4_] relative to the crown ether ring. (d) and (f) Schematic diagram of rotation and calculated rotational energy barrier diagram for the methylated cyclohexylamine molecule in the structure [(CH_3_-C_6_H_10_-NH_3_)(18-crown-6)][ClO_4_] relative to the crown-ether ring. It is evident that the rotational energy barrier in the methylated molecular structure is lower than in the cyclohexylamine structure. This implies that the molecule can overcome the rotational barrier at lower temperatures, transitioning from an ordered state to a rotationally disordered state, thereby inducing a structural phase transition.


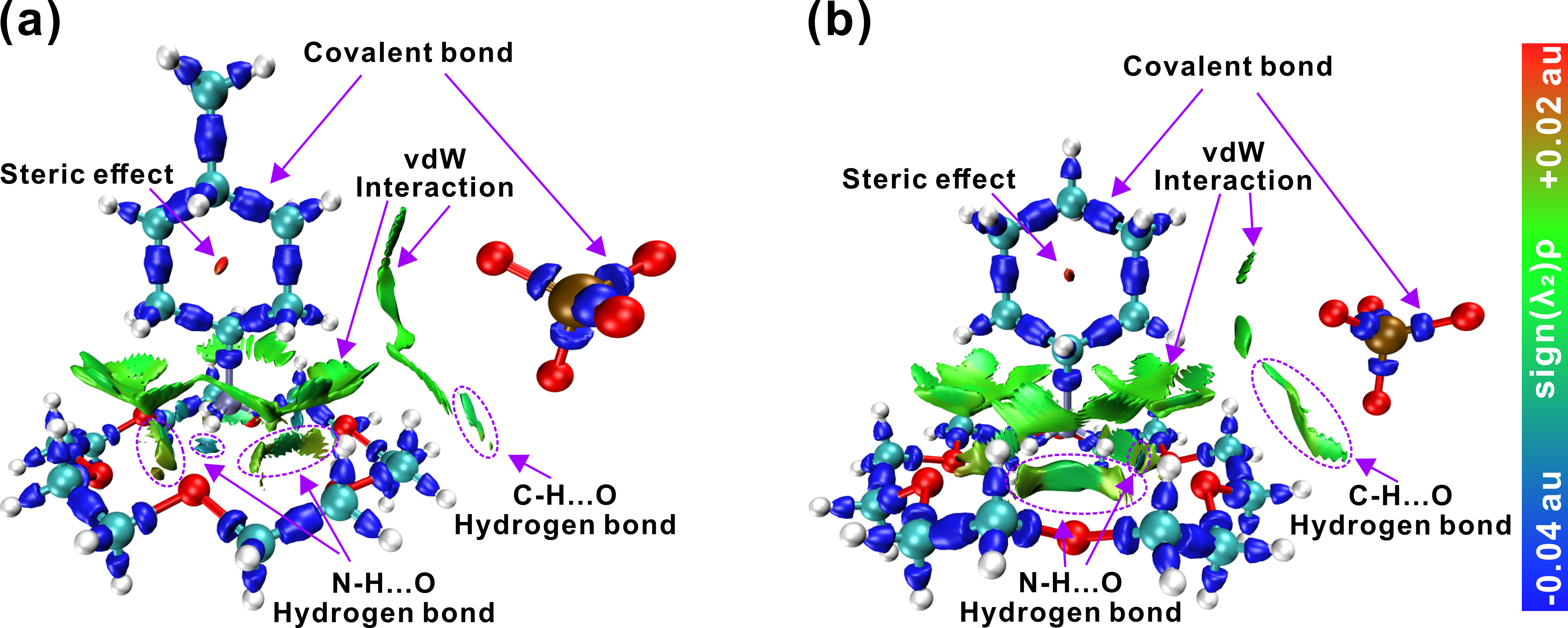


Figure S6. Isosurface maps of (a) [(CH_3_-C_6_H_10_-NH_3_)(18-crown-6)][ClO_4_] and (b) [(C_6_H_11_-NH_3_)(18-crown-6)][ClO_4_], showing hydrogen bonding interactions.


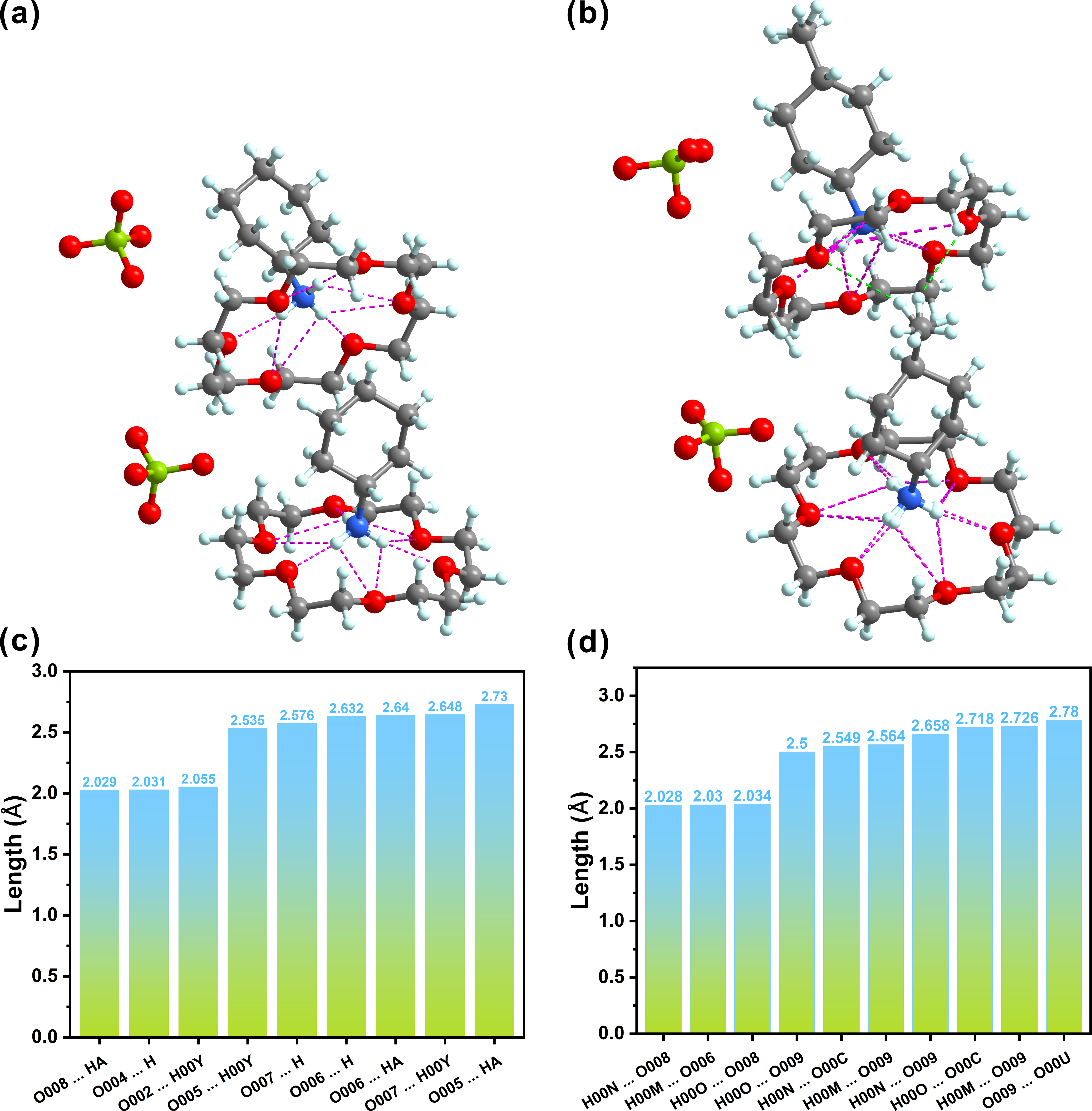


Figure S7. (a) and (c) Hydrogen bond network formed between cyclohexylamine molecules and surrounding functional crown-ether rings in the structure [(C_6_H_11_-NH_3_)(18-crown-6)][ClO_4_] and the bond lengths of asymmetric hydrogen bond. (b) and (d) Hydrogen bond network and asymmetric hydrogen bond bond lengths formed between the methylated cyclohexylamine molecules and the surrounding crown-ether rings in the structure [(CH_3_-C_6_H_10_-NH_3_)(18-crown-6)][ClO_4_]; It is evident that new hydrogen bonds exist between CH_3_- and O atoms on the crown ether rings, and the hydrogen bond network density is significantly enhanced, which will promote higher thermal stability of the structure.


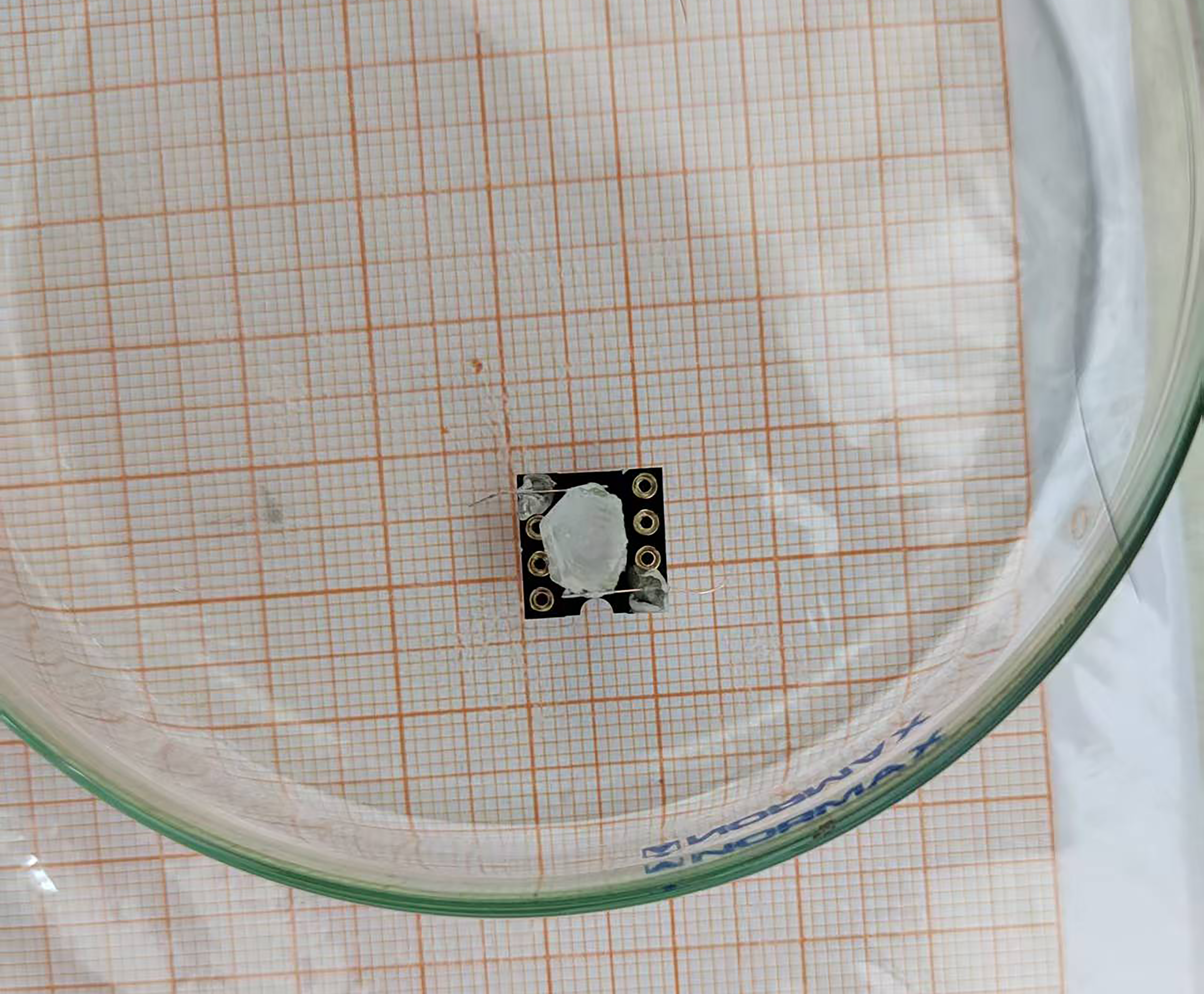


Figure S8. The photographs of [(CH_3_-C_6_H_10_-NH_3_)(18-crown-6)][ClO_4_] actual samples used for P-E measurements along the [001] axis.


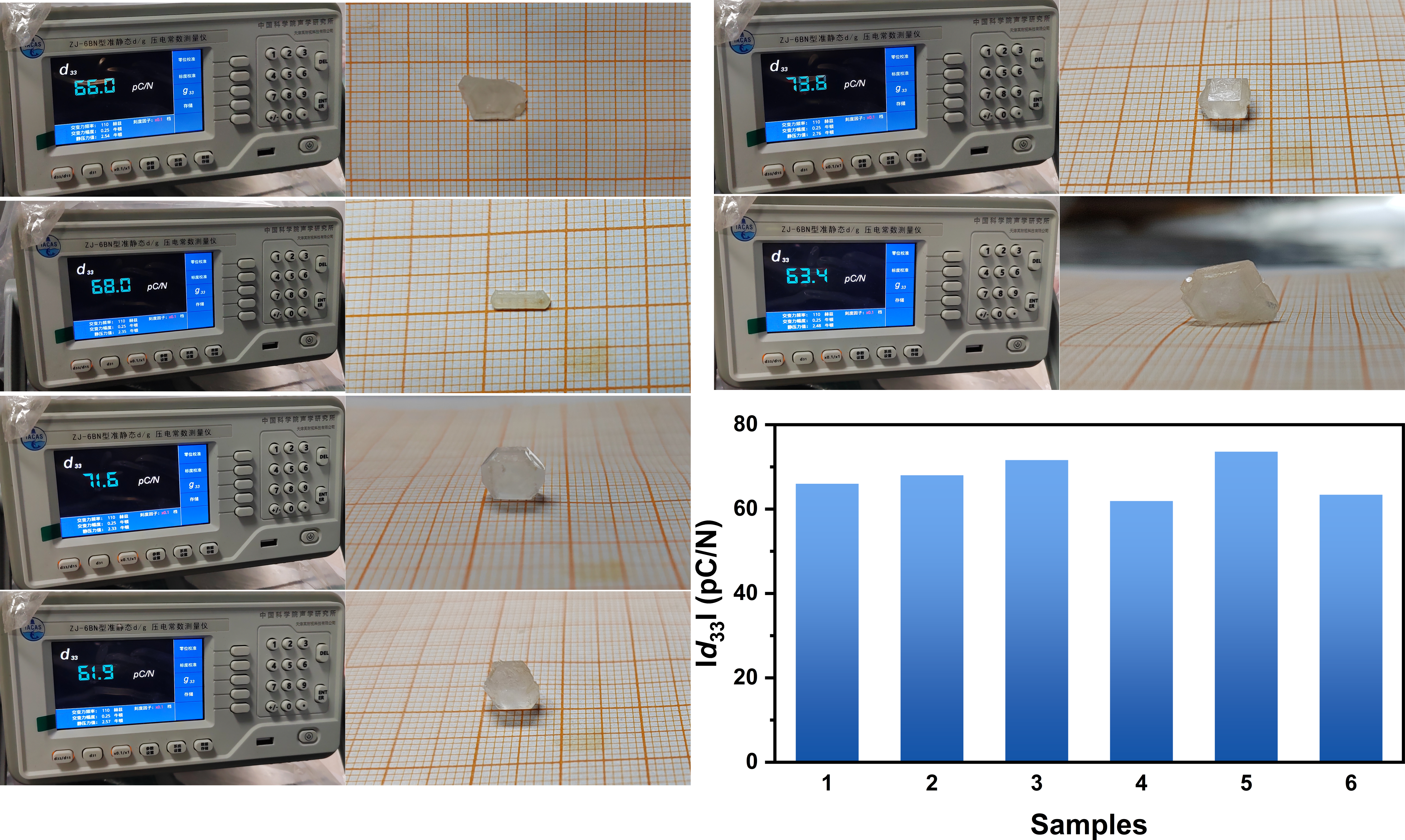


Figure S9. Piezoelectric $d_{33}$ data of [(CH_3_-C_6_H_10_-NH_3_)(18-crown-6)][ClO_4_] single crystals.


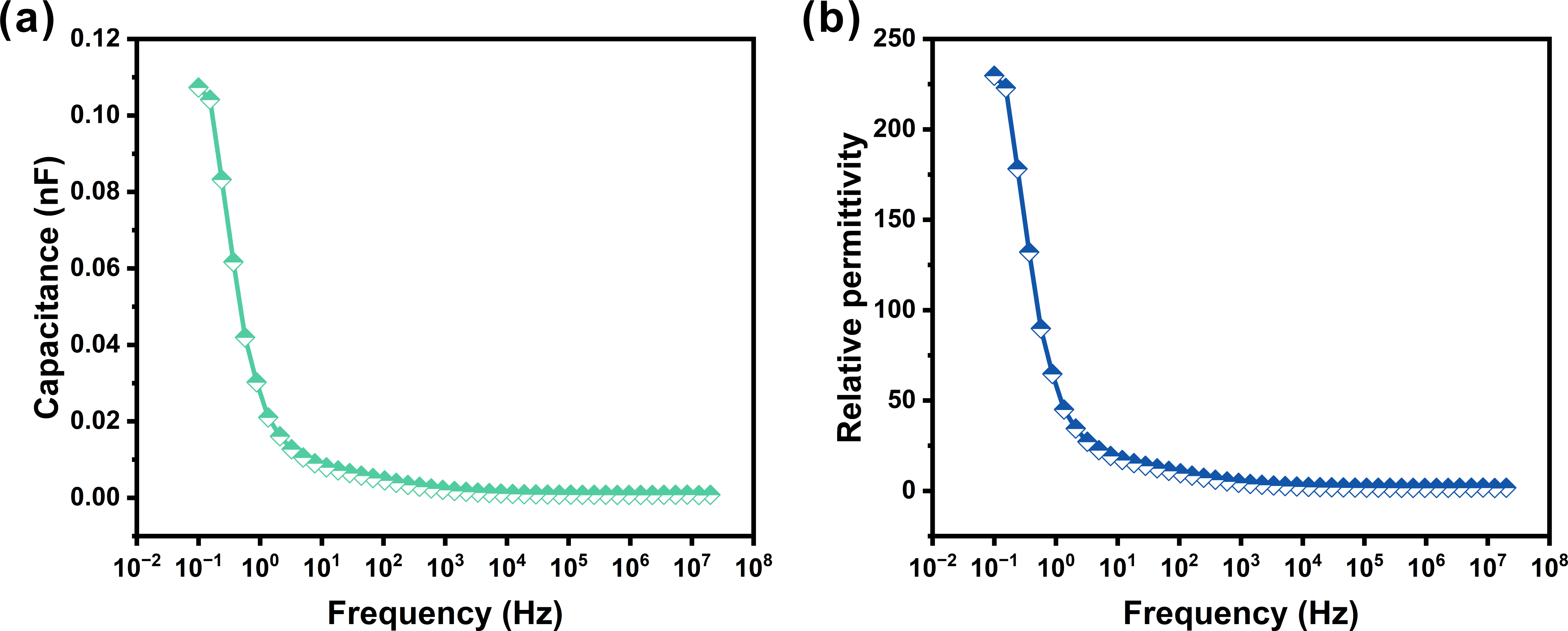


Figure S10. (a) Frequency dependent capacitance tested from 0.1 Hz to 20 MHz of [(CH_3_-C_6_H_10_-NH_3_)(18-crown-6)][ClO_4_] crystalline powder . (b) Relative dielectric permittivity (${}^{'}$) as a function of frequency.


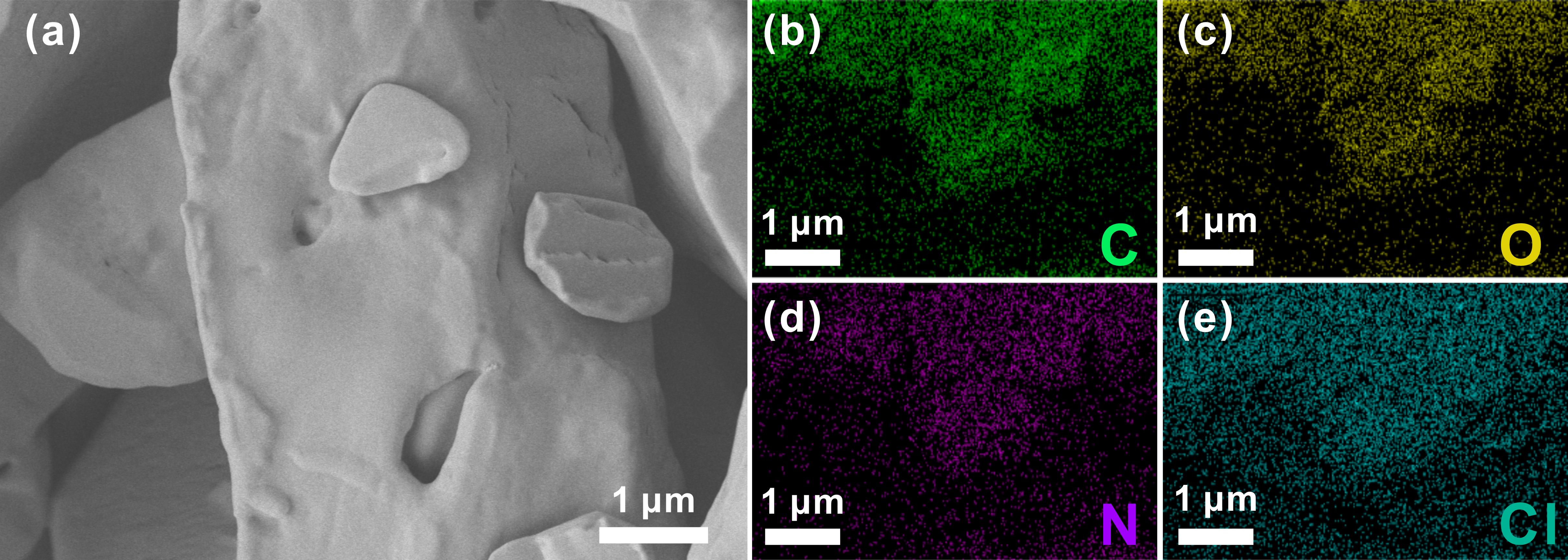


Figure S11. (a) FE-SEM micrograph and (b-e) EDS mapping images of the 50 wt% [(CH_3_-C_6_H_10_-NH_3_)(18-crown-6)][ClO_4_]/TPU composite.





Figure S12. Dielectric frequency response of a 50 wt% [(CH_3_-C_6_H_10_-NH_3_)(18-crown-6)][ClO_4_]/TPU composite at 300 K. (a) Frequency dependence of the real part of the dielectric permittivity ${}^{'}$; (b) Frequency dependence of the imaginary dielectric permittivity ${}^{''}$; (c) Frequency dependence of the tangent of the loss angle tanδ; (d) Frequency dependence of the conductivity $\sigma^{'}$; (e) Relationship between the imaginary dielectric permittivity ${}^{''}$ and the real dielectric permittivity ${}^{'}$ (Cole-Cole plot).

Dielectric-frequency characterization was performed on a 50 wt% [(CH₃-C₆H₁₀-NH₃)(18-crown-6)] [ClO₄]/TPU non-dense composite. The wide-frequency dielectric spectrum at room temperature (0.1 Hz–20 MHz) indicates that both the real part of the dielectric permittivity (${}^{'}$) and the imaginary part (${}^{''}$) remain stable in the high-frequency region (f > 10⁶ Hz). ${}^{'}$ values range between 150–155, while ${}^{''}$ is extremely low. This is primarily attributed to intrinsic electric atom/electron polarization, which is less affected by microstructural voids in the film ^[^[^3^](#_ENREF_4)^]^. In the low-to-mid-frequency range (f < 10⁶ Hz), ${}^{'}$ increases sharply with decreasing frequency, reaching a 294% increase, while ${}^{''}$ also significantly increases. This phenomenon is primarily driven by hetero-interface polarization formed between voids within the film and the polymer matrix. This polarization is superimposed on the ferroelectric intrinsic polarization, elevating ${}^{'}$ to approximately 157.2 at 1 kHz. This provides the necessary polarization strength to enhance the piezoelectric response ^[^[^3-4^](#_ENREF_4)^]^. Given that data above 1 MHz is affected by parasitic parameters, subsequent analysis focuses on the reliable frequency range below 1 MHz ^[^[^5^](#_ENREF_6)^]^. The tangent of the loss angle (anδ) and ${}^{''}$ exhibit synchronous, dramatic increases in the low-to-mid frequency range, with tanδ reaching 16.37 at the lowest frequency. This loss originates from the superposition of interfacial polarization loss and leakage conductance loss: interfacial polarization loss stems from the resistance to charge accumulation and migration at the heterointerface, directly manifesting as an increase in ${}^{''}$; while leakage conductance loss arises from conductive pathways formed by voids and non-ideal electrode contacts, further amplifying ${}^{''}$ ^[^[^5-6^](#_ENREF_6)^]^. The corresponding conductivity ($\sigma^{'}$) decreases slowly at low frequencies, indicating that charges tend to accumulate locally at the interface rather than undergo long-range migration ^[^[^3^](#_ENREF_4)^]^. The Cole-Cole plot (${}^{''}$ vs. ${}^{'}$) exhibits an approximately linear upward trend rather than the typical Debye relaxation semicircle. This indicates that the low-frequency region is dominated by space-charge polarization (interface polarization and leakage conductance), whose strong ${}^{''}$ response masks the characteristic peak of the ferroelectric intrinsic dipole relaxation ^[^[^3^](#_ENREF_4)^,^ [^7^](#_ENREF_8)^]^. Fitting analysis confirms dual polarization mechanisms at room temperature: near-ideal Debye-type dipole relaxation in the mid-frequency range, facilitating rapid piezoelectric response; and interface polarization dominating the low-frequency range, serving as the core mechanism for polarization enhancement but accompanied by significant ${}^{''}$ loss ^[^[^3-4^](#_ENREF_4)^]^.





Figure S13. Dielectric temperature spectra of 50 wt% [(CH_3_-C_6_H_10_-NH_3_)(18-crown-6)][ClO_4_]/TPU composite. (a–i) Temperature dependence of the real part of the dielectric constant ${}^{'}$, the imaginary part ${}^{''}$, and the tangent of the loss angle tanδ at 0.1 Hz, 1 kHz, and 1 MHz, respectively.

The temperature-frequency spectra of ${}^{'}$ and ${}^{''}$ further reveal the thermal activation characteristics of interfacial polarization. In the low-temperature region (-60 to -20 ℃), thermal motion is weak, suppressing interfacial polarization, with ${}^{'}$ and ${}^{''}$ remaining stable across all frequencies (fluctuations <3%). As temperature increases to the intermediate range (-20 to 20 ℃) and high-temperature range (20 to 60 ℃), ${}^{'}$ and ${}^{''}$ at low frequencies are significantly activated, exhibiting exponential growth particularly at 0.1 Hz, while high-frequency responses remain stable ^[^[^3^](#_ENREF_4)^,^ [^7^](#_ENREF_8)^]^. This behavior exhibits strong correlation with the temperature evolution of tanδ, indicating that the temperature dependence of interfacial polarization directly governs the thermal stability of piezoelectric performance ^[^[^4^](#_ENREF_5)^,^ [^6^](#_ENREF_7)^]^. Experiments demonstrate that in this non-dense composite, the polarization induced by void interfaces effectively superimposes with the intrinsic polarization of the ferroelectric material, significantly enhancing the macroscopic polarization strength (manifested as increased ${}^{'}$). This provides a key pathway for optimizing the piezoelectric coefficient ^[^[^4^](#_ENREF_5)^,^ [^6^](#_ENREF_7)^]^. However, the loss characteristics reflected by the synchronous increase of ${}^{''}$ with temperature elevate the electromechanical conversion efficiency and compromise the stability of piezoelectric performance ^[^[^6^](#_ENREF_7)^,^ [^8^](#_ENREF_9)^]^.


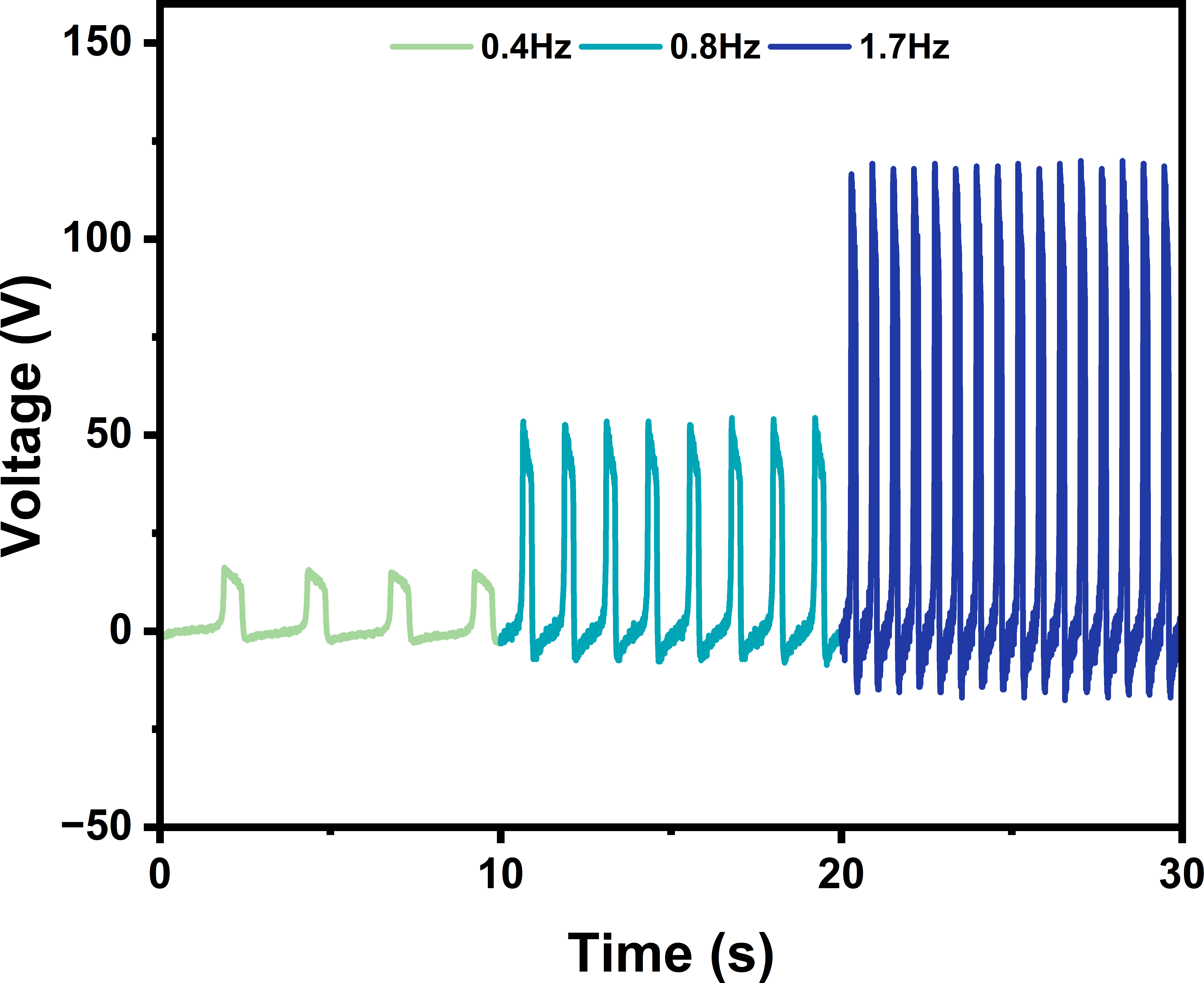


Figure S14. Frequency-dependent output voltage of 50 wt% [(CH_3_-C_6_H_10_-NH_3_)(18-crown-6)][ClO_4_]/TPU devices under a compressing force of 150 N.


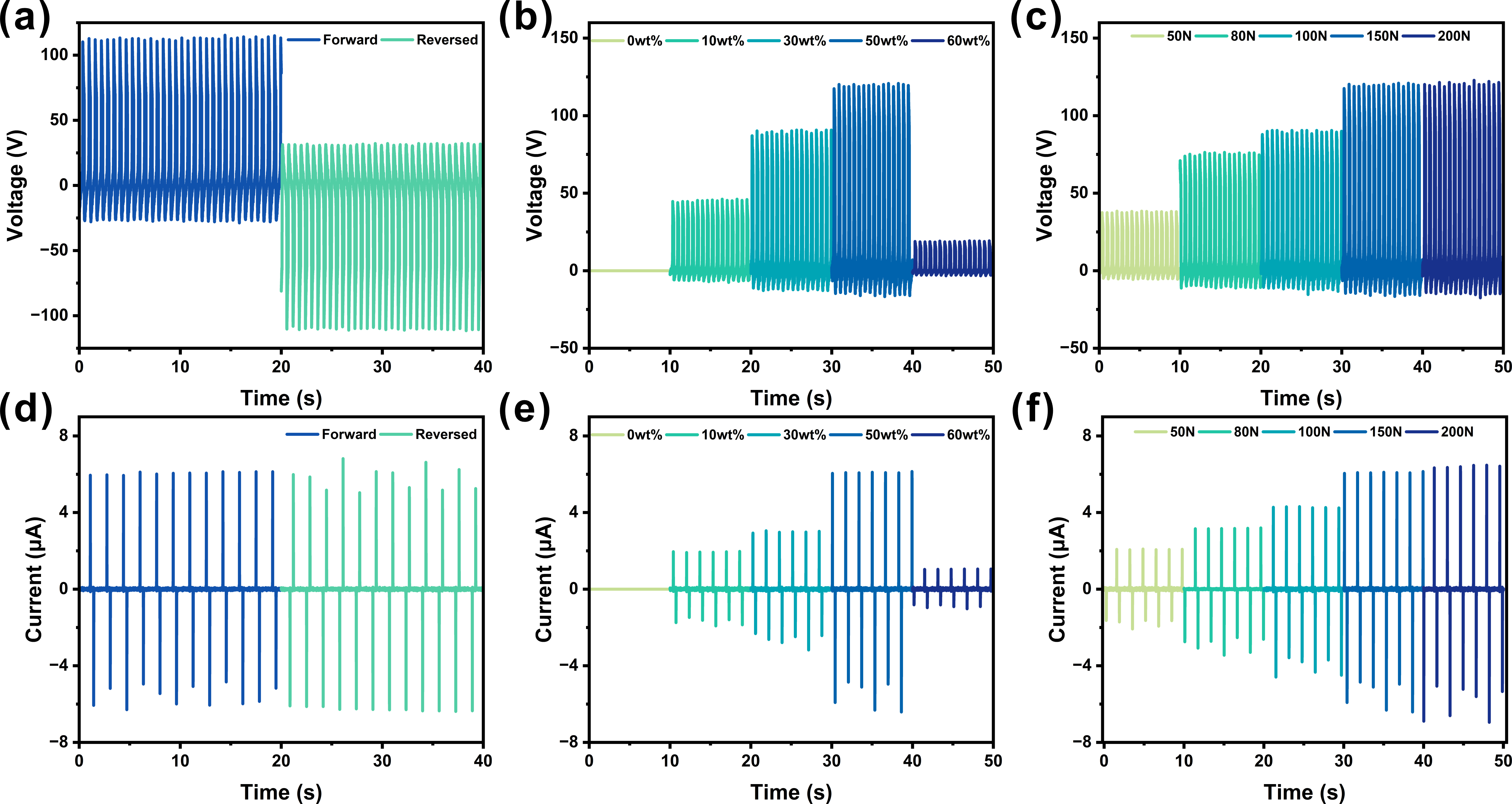


Figure S15. (a) and (d) The polarization switching output voltage (*V*_oc_) and short-circuit current (*I*_sc_) of 50% [(CH_3_-C_6_H_10_-NH_3_)(18-crown-6)][ClO_4_]/TPU composite devices with 150 N (1.7 Hz). (b) and (e) The piezoelectric energy harvester output *V*_oc_ and *I*_sc_ signals of the piezoelectric energy harvesters with different contents. (c) and (f) *V*_oc_ and *I*_sc_ of piezoelectric energy harvester output for 50% composite films under applying force from 50 to 200 N.


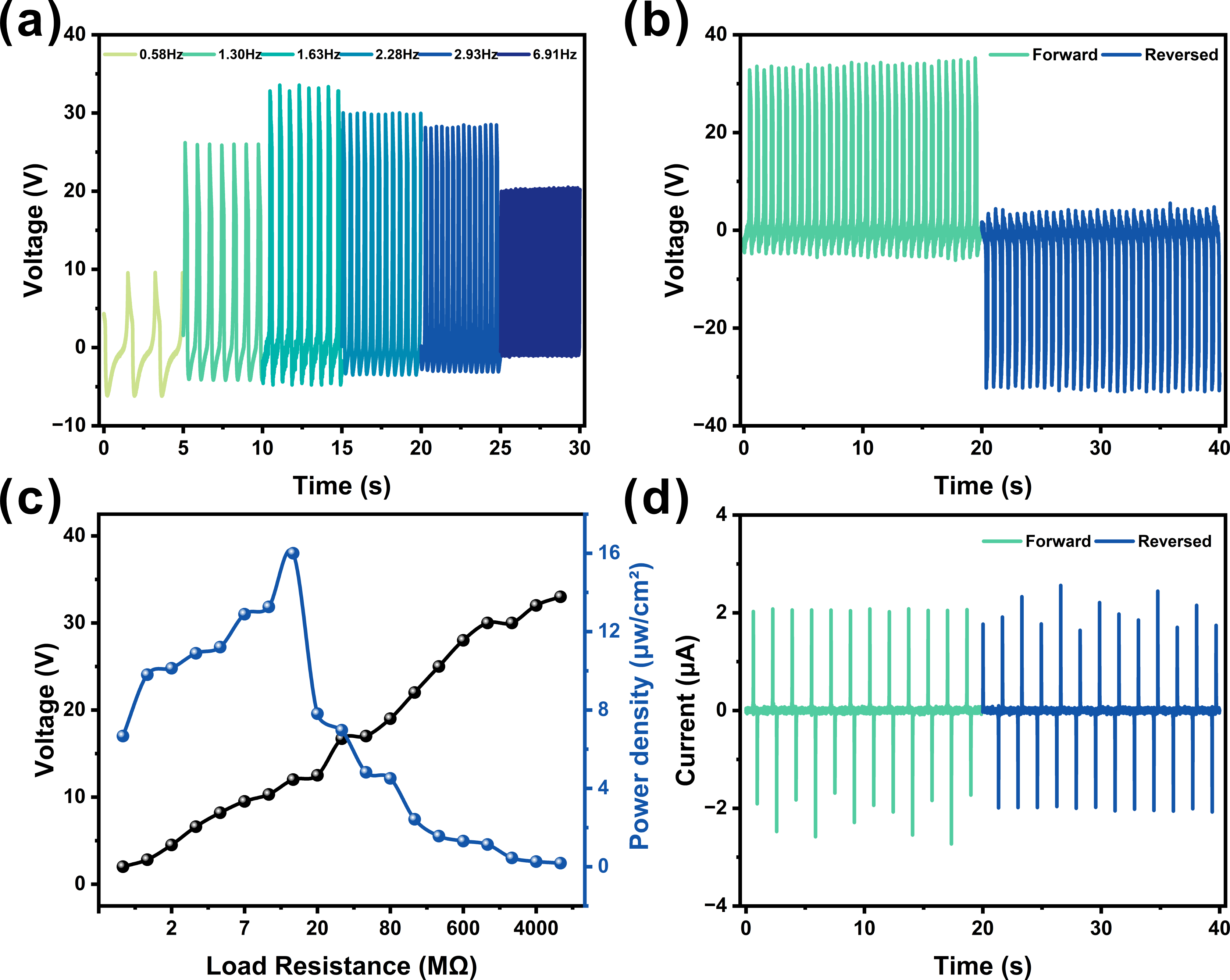


Figure S16. Piezoelectric energy harvesting performance of 50% [(CH_3_-C_6_H_10_-NH_3_)(18-crown-6)][ClO_4_]/TPU composite devices after 8 months of exposure under 50 N. (a) Frequency-dependent output voltage (*V*_oc_). (c) *V*_oc_ and power density under load-resistance. (b) and (d) The polarization switching *V*_oc_ and short-circuit current (*I*_sc_) (1.63 Hz).


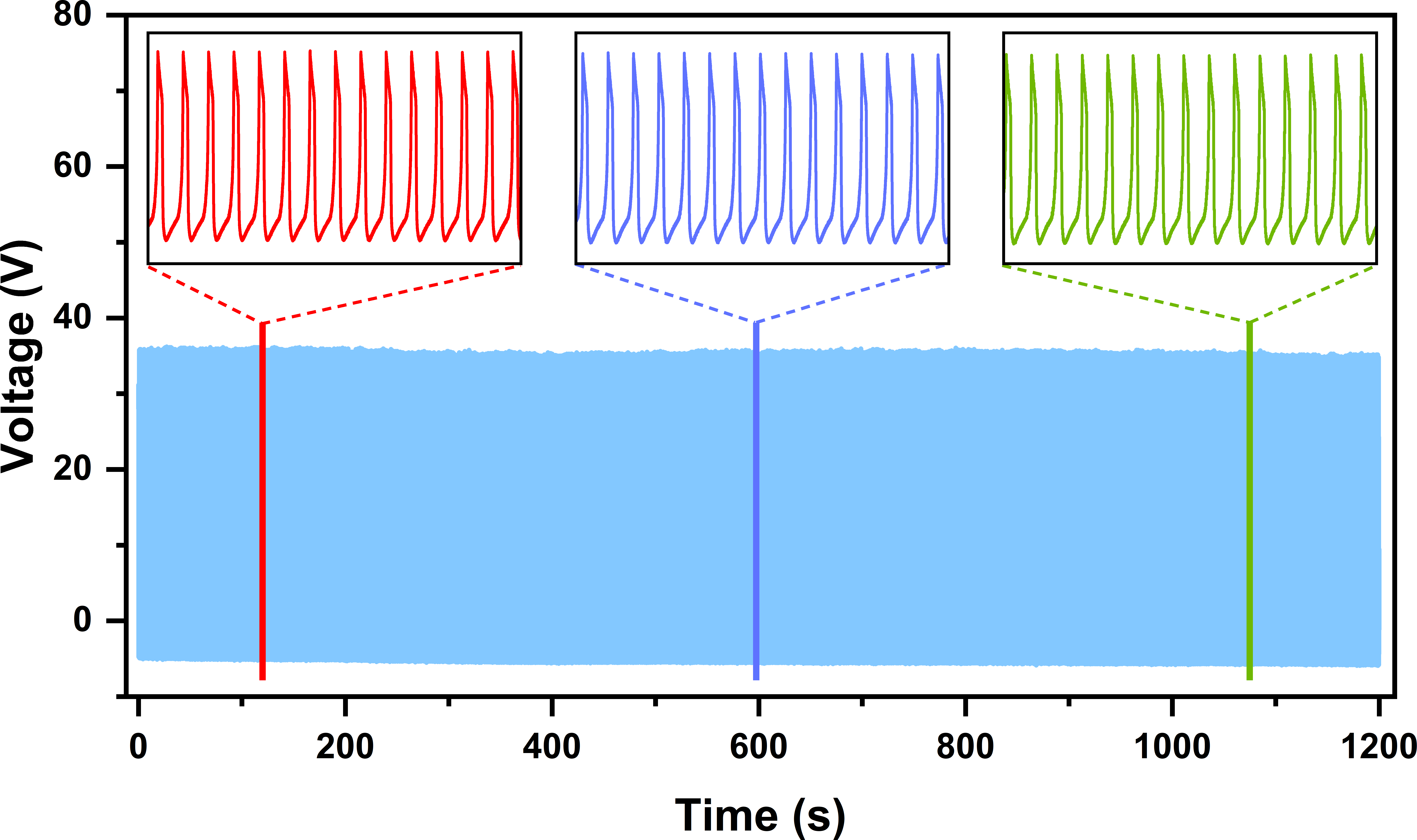


Figure S17. Long-term stability of 50% [(CH_3_-C_6_H_10_-NH_3_)(18-crown-6)][ClO_4_]/TPU composite devices under 50 N (1.63 Hz) for 8 months placement. The output open circuit voltage *V*_oc_ were retained during 1200 s without fluctuation, demonstrating the excellent stability.


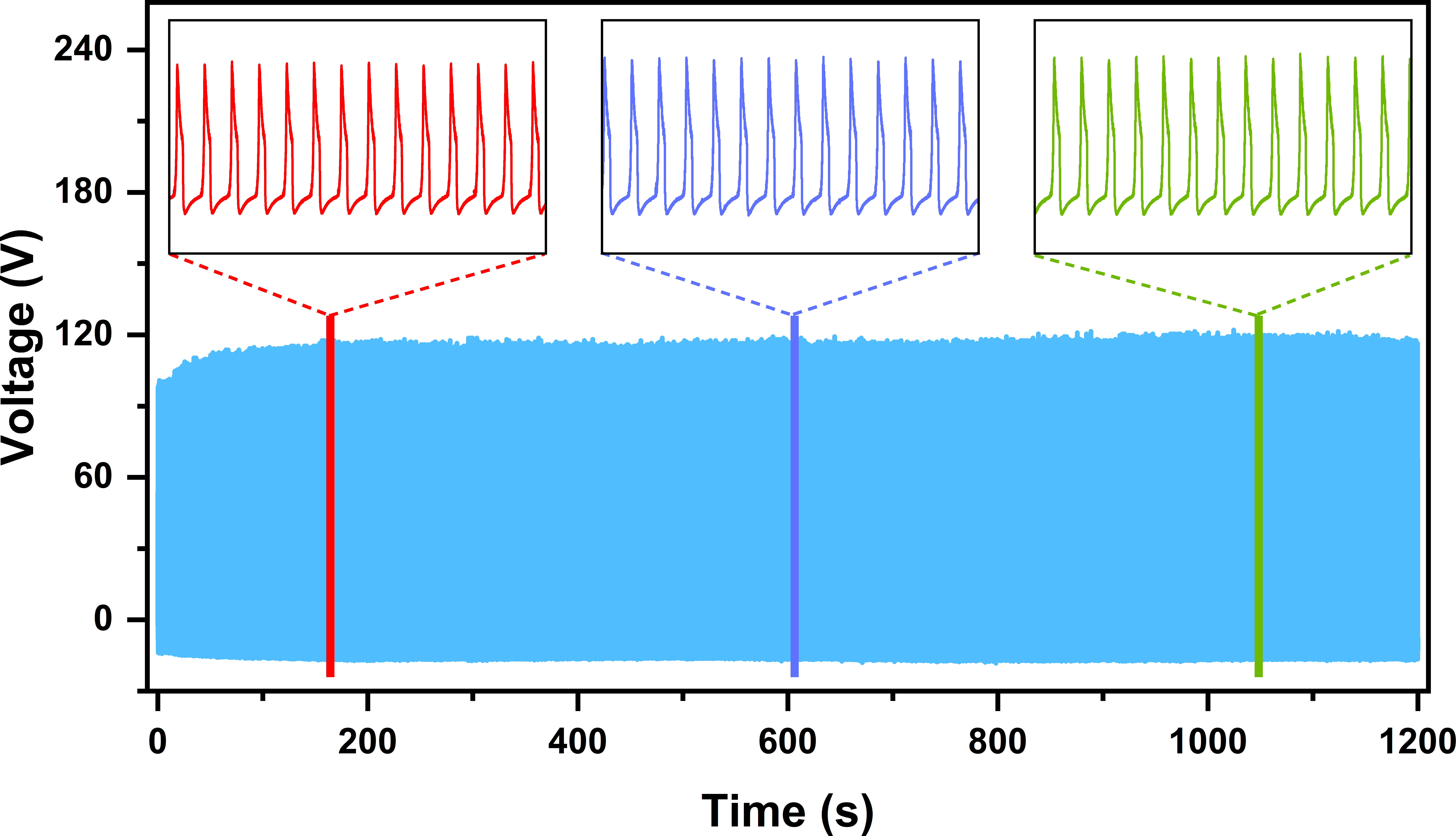


Figure S18. Long-term stability of 50% [(CH_3_-C_6_H_10_-NH_3_)(18-crown-6)][ClO_4_]/TPU composite devices under 150 N (1.7 Hz) for 8 months placement. The output open circuit voltage *V*_oc_ were retained during 1200 s without fluctuation, demonstrating the excellent stability.


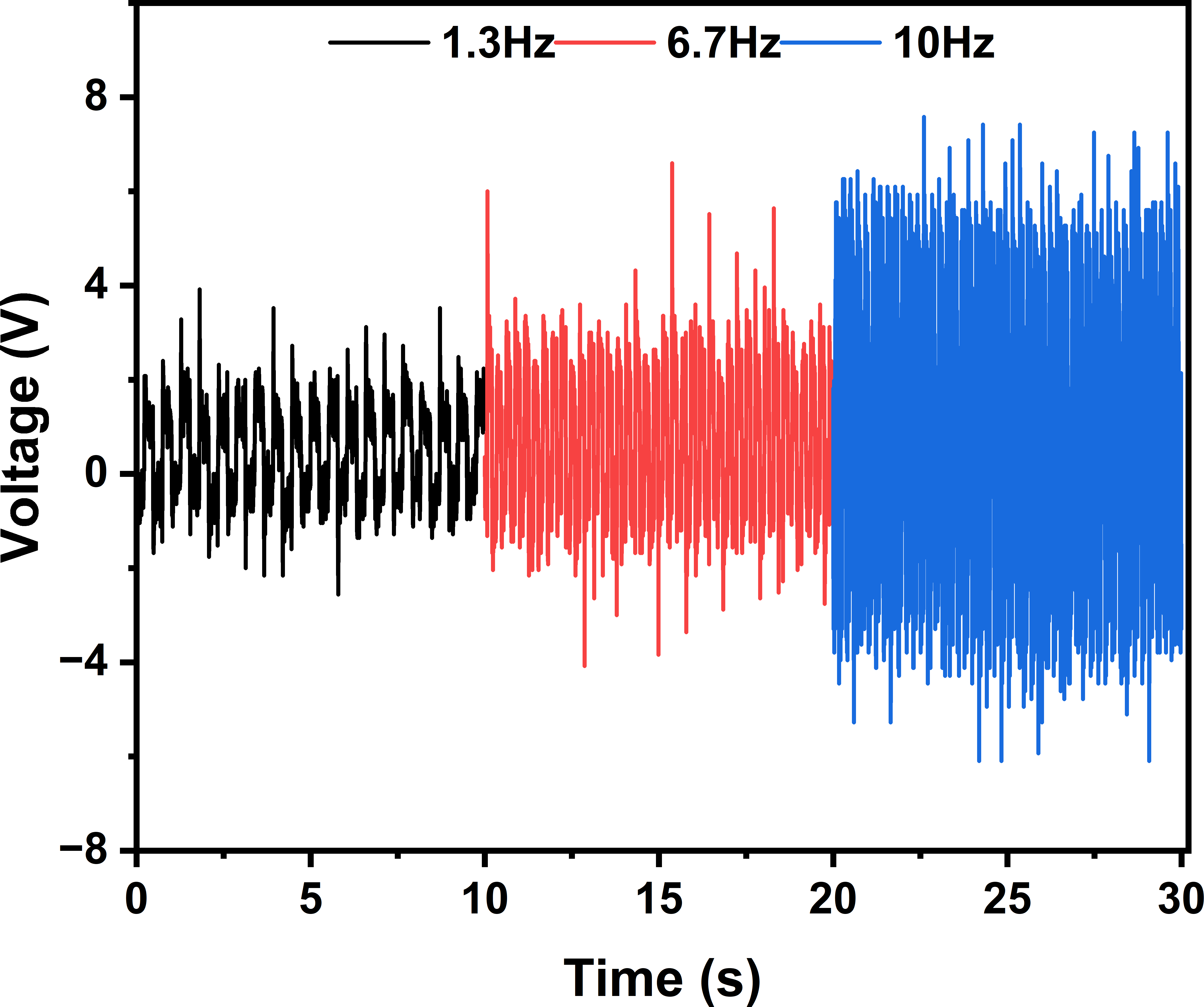


Figure S19. Frequency-dependent output voltage of 1 wt% [(CH_3_-C_6_H_10_-NH_3_)(18-crown-6)][ClO_4_]/PDMS devices under a compressing force of 50 N.


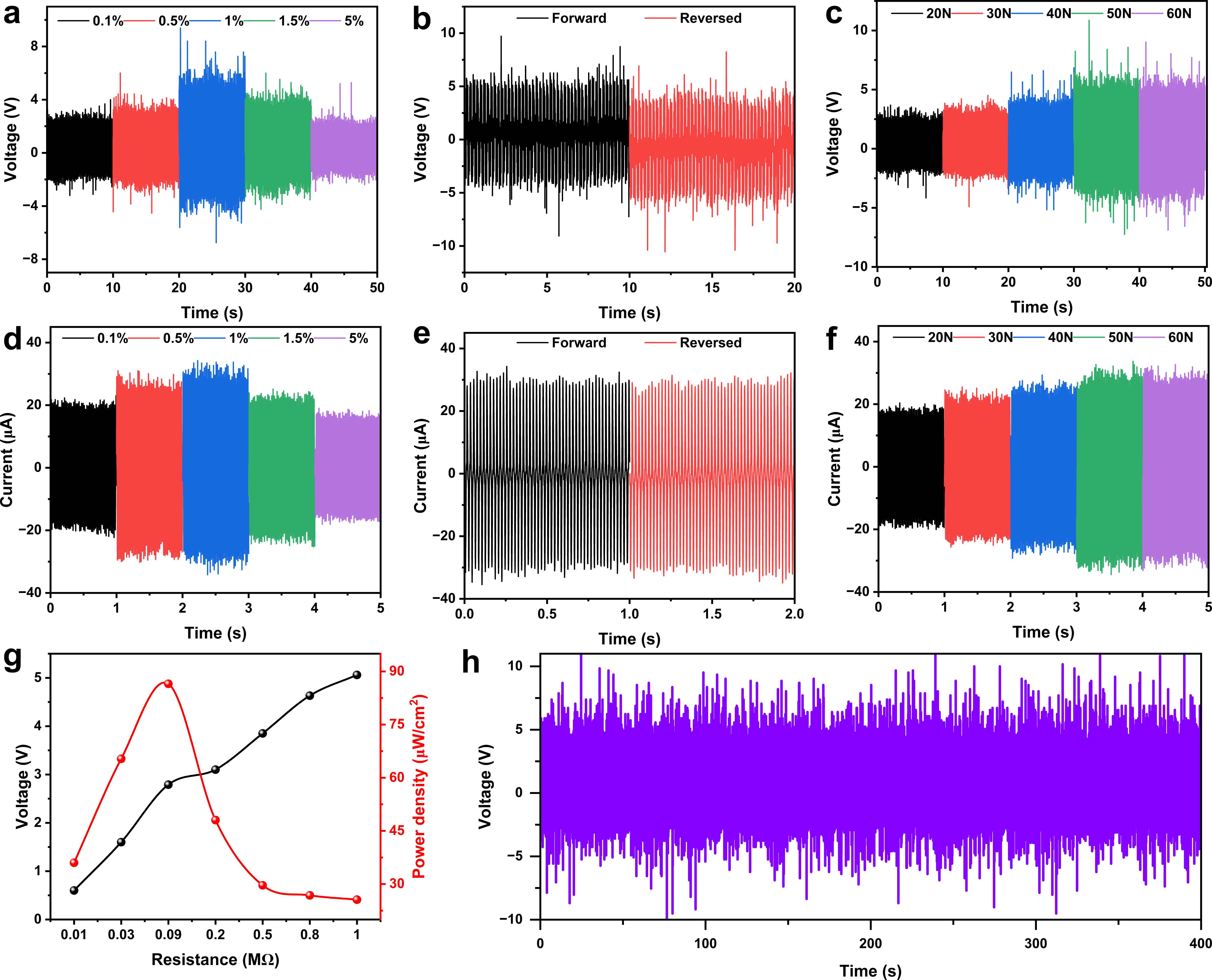


Figure S20. Piezoelectric energy harvesting performance of [(CH_3_-C_6_H_10_-NH_3_)(18-crown-6)][ClO_4_]/PDMS composite films. (a) and (d) The piezoelectric energy harvester output voltage (*V*_oc_) and short-circuit current (*I*_sc_) signals of [(CH_3_-C_6_H_10_-NH_3_)(18-crown-6)][ClO_4_] piezoelectric energy harvesters with different contents. The polarization switching output (b) *V*_oc_ and (e) *I*_sc_ of 1% [(CH_3_-C_6_H_10_-NH_3_)(18-crown-6)][ClO_4_]/PDMS composite film with 50 N (10 Hz). (c) *V*_oc_ and (f) *I*_sc_ of piezoelectric energy harvester output for 1% [(CH_3_-C_6_H_10_-NH_3_)(18-crown-6)][ClO_4_]/PDMS composite films under applying force from 20 to 60 N. (g) The resistance-dependent voltages and power densities under 50 N. (h) The long-term output performance tested for 400 seconds.


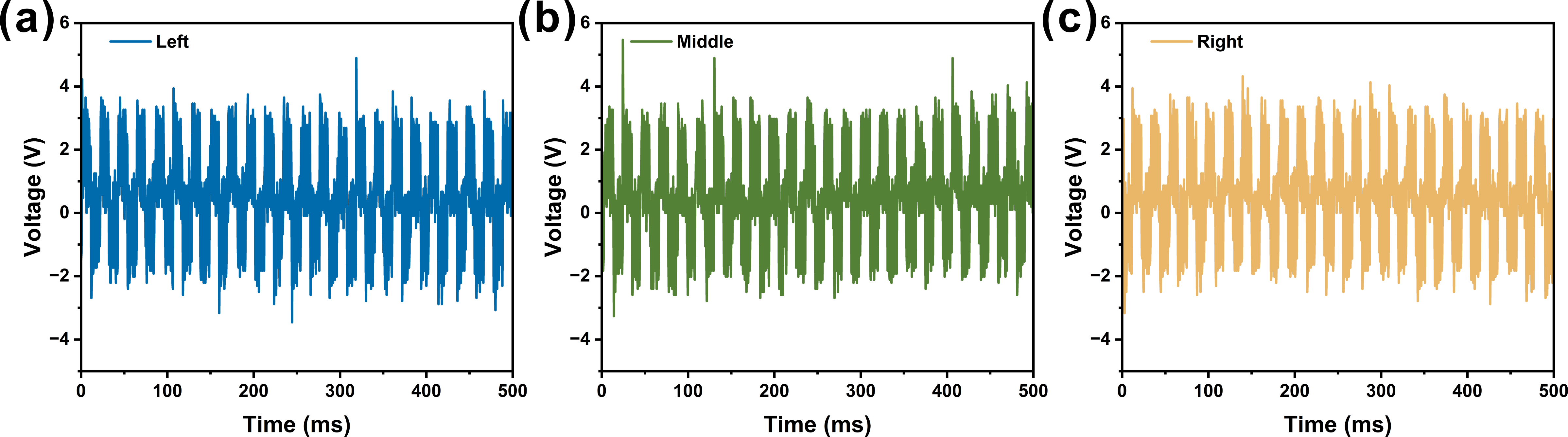


Figure S21. Long-term stability of 50% [(CH_3_-C_6_H_10_-NH_3_)(18-crown-6)][ClO_4_]/TPU underwater ultrasonic composite devices for 8 months placement. The output open circuit voltage *V*_oc_ were retained during 500 s without fluctuation, demonstrating the excellent stability.

Calculation of polarization of [(CH_3_-C_6_H_10_-NH_3_)(18-crown-6)][ClO_4_] according to a point charge model


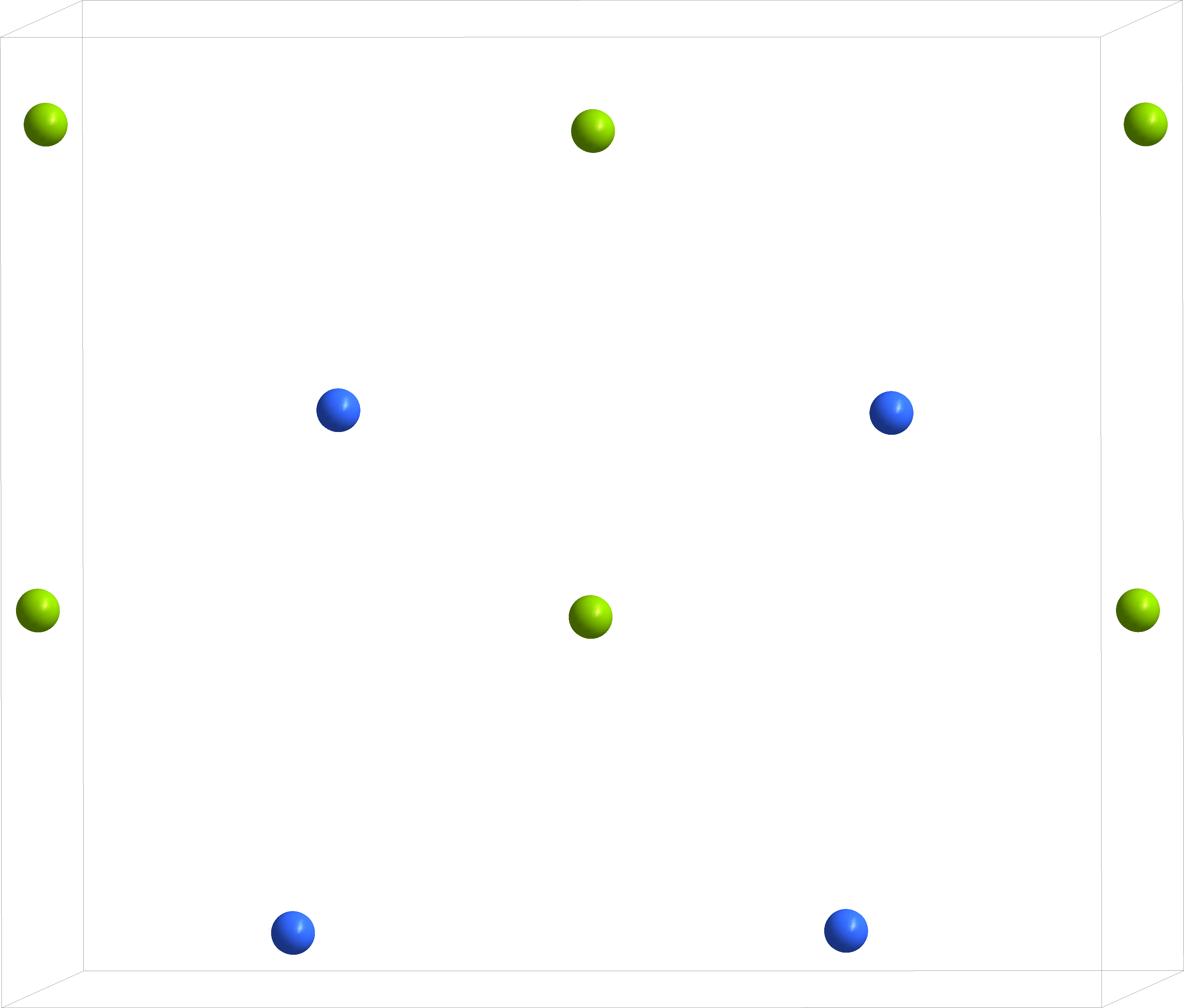


Cl2^2^

Cl1^4^

Cl1^2^

Cl2^1^

Cl1^3^

Cl1^1^

N2^1^

N1^1^

N1^2^

N2^2^

Figure S22. Distribution of Cl and N atoms of [(CH_3_-C_6_H_10_-NH_3_)(18-crown-6)][ClO_4_] in a unit cell at 100 K.


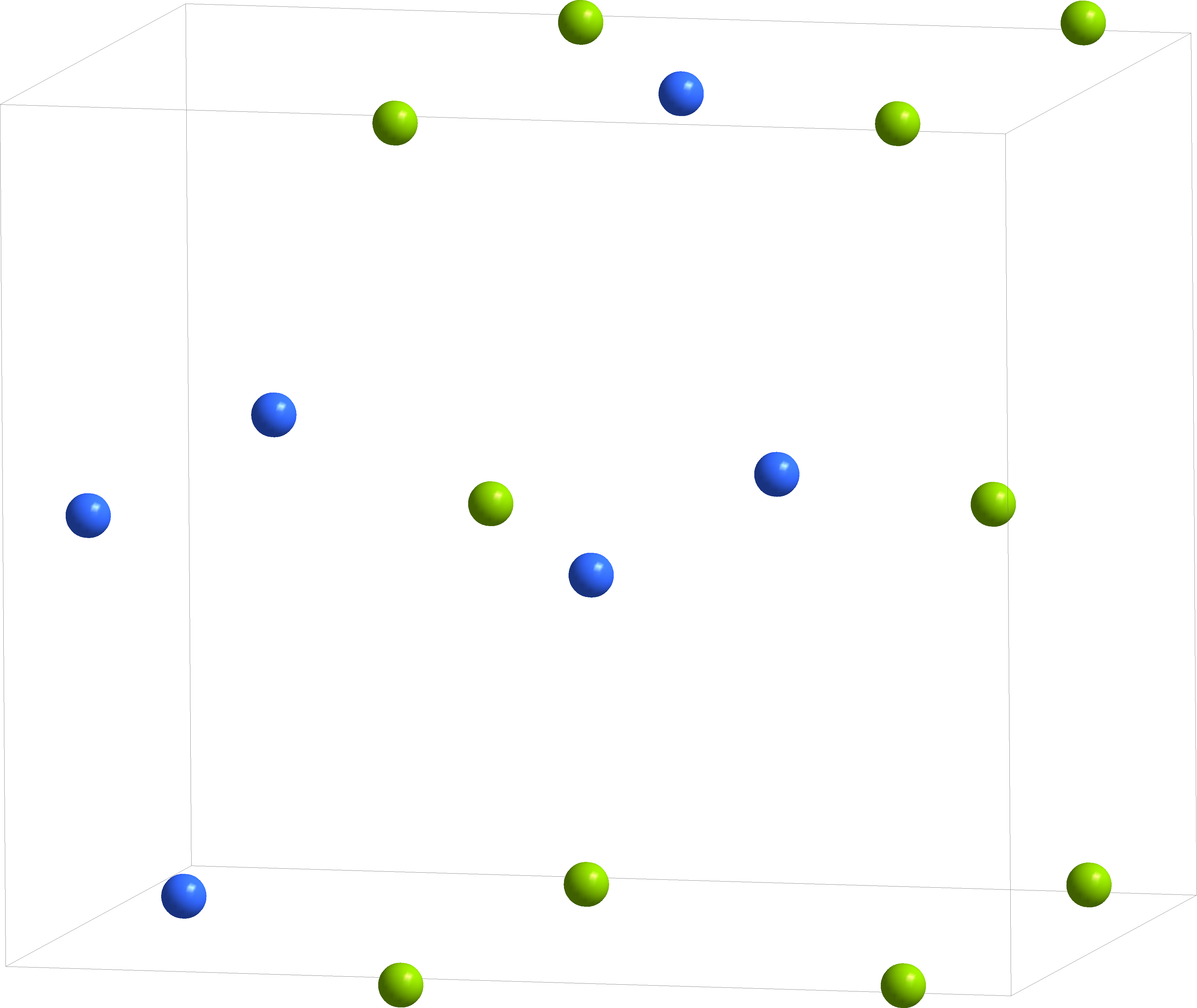


Cl1^1^

N1^6^

N1^5^

Cl1^10^

Cl1^9^

Cl1^8^

Cl1^7^

Cl21^6^

Cl1^5^

N1^4^

N1^3^

N1^2^

N1^1^

Cl1^4^

Cl1^3^

Cl1^2^

Figure S23. Distribution of Cl and N atoms of [(CH_3_-C_6_H_10_-NH_3_)(18-crown-6)][ClO_4_] in a unit cell at 293 K.

Table S1. Crystallographic Data and Structure Refinements for [(C_6_H_11_-NH_3_)(18-crown-6)][ClO_4_] and [(CH_3_-C_6_H_10_-NH_3_)(18-crown-6)][ClO_4_].

|  | [(C_6_H_11_-NH_3_)(18-crown-6)][ClO_4_] | [(CH_3_-C_6_H_10_-NH_3_)(18-crown-6)][ClO_4_] | |
| --- | --- | --- | --- |
| Temperature | 293 K | 100 K | 293 K |
| Empirical formula | [(C_6_H_11_-NH_3_)(18-crown-6)][ClO_4_] | [(CH_3_-C_6_H_10_-NH_3_)(18-crown-6)][ClO_4_] | |
| Formula weight | 463.94 | 477.97 | 477.97 |
| Crystal system | orthorhombic | monoclinic | orthorhombic |
| Space group | *Pca*2_1_ | *P*2_1_ | *Cmc*2_1_ |
| *a*/Å | 13.2932(9) | 8.7679(3) | 11.4826(12) |
| *b*/Å | 12.2716(9) | 15.5060(7) | 13.4466(16) |
| *c*/Å | 14.2179(9) | 17.5328(7) | 16.0371(18) |
| *α/ ◦* | 90 | 90 | 90 |
| *β/ ◦* | 90 | 97.581(4) | 90 |
| *γ/ ◦* | 90 | 90 | 90 |
| Volume (Å^3^) | 2319.3(3) | 2362.84(17) | 2476.2(5) |
| *Z* | 4 | 4 | 4 |
| *R*_int_ | 0.0201 | 0.0272 | 0.0281 |
| GOF | 1.033 | 1.061 | 1.261 |
| *R*_1_ [*I* > 2*σ*(*I*)] | 0.0410 | 0.0619 | 0.1094 |
| *wR*_2_ [*I* > 2*σ*(*I*)] | 0.1191 | 0.1672 | 0.3321 |

Table S2. Coordinate positions of Cl atoms and the surrounding N atoms of [(CH_3_-C_6_H_10_-NH_3_)(18-crown-6)][ClO_4_] at 100 K. According to the crystal structure data collected at 100 K, we select a unit cell and assume that the centers of the positive charges and the negative charges are located on the N atoms and Cl atoms, respectively.

| **Atoms** | **Atom coordinate** | | **Coordinate of charge center** |
| --- | --- | --- | --- |
| N | N1^1^(0.01942, 0.42158, 0.23371) | N1^2^(0.9806, 0.92158, 0.76631) | (0.5, 0.67285, 0.5) |
|  | N2^1^(0.03169, 0.42408, 0.73744) | N2^2^(0.96833, 0.92408, 0.26258) |  |
| Cl | Cl1^1^(0.49171, 0.10932, 0.00324) | Cl1^2^(0.50831, 0.60932, -0.00323) | (0.5, 0.36157, 0.5) |
|  | Cl1^3^(0.49171, 0.10932, 1.00324) | Cl1^4^(0.50831, 0.60932, 0.99677) |  |
|  | Cl2^1^(0.49024, 0.11604, 0.50068) | Cl2^2^(0.50978, 0.61604, 0.49934) |  |

The cell parameters: *a* = 8.7679(3) Å, *b* = 15.5060(7) Å, *c* = 17.5328(7) Å, *β* = 97.581(4)°, *V* = 2362.84(17) Å^3^ and *Z* = 4.

$$P_{s}=lim\frac{1}{V}\sum q_{i}r_{i}$$

$$=\frac{1.602*{10}^{-19}C*(0.67285-0.36157)*{4*15.5060*10}^{-10} m}{2362.84*{10}^{-30}m^{3}}$$

$$=0.13089986 C m^{-2}$$

$$\left| P_{s} \right|=13.09 \mu C \mathrm{cm}^{-2}$$

Table S3. Coordinate positions of Cl atoms and the surrounding N atoms of [(CH_3_-C_6_H_10_-NH_3_)(18-crown-6)][ClO_4_] at 293 K. According to the crystal structure data collected at 293 K, we select a unit cell and assume that the centers of the positive charges and the negative charges are located on the N atoms and Cl atoms, respectively.

| **Atoms** | **Atom coordinate** | | **Coordinate of charge center** |
| --- | --- | --- | --- |
| N | N1^1^(0.00001, 0.47401, 0.41499) | N1^2^(1.00001, 0.47401, 0.41499) | (0.5,0.5, 0.6650) |
|  | N1^3^(0.5, 0.97401, 0.41499) | N1^4^(0.00001, 0.52601, 0.91499) |  |
|  | N1^5^(1.00001, 0.52601, 0.91499) | N1^6^(0.5, 0.02601, 0.91499) |  |
| Cl | Cl1^1^(0.5, 0.49183, 0.60713) | Cl1^2^(0.00001, -0.00817, 0.60713) | (0.5,0.5, 0.3571) |
|  | Cl1^3^(1.00001, -0.00817, 0.60713) | Cl1^4^(0.00001, 0.99183, 0.60713) |  |
|  | Cl1^5^(1.00001, 0.99183, 0.60713) | Cl1^6^(0.5, 0.50819, 0.10713) |  |
|  | Cl1^7^(0.00001, 0.00819, 0.10713) | Cl1^8^(1.00001, 0.00819, 0.10713) |  |
|  | Cl1^9^(0.00001, 1.00819, 0.10713) | Cl1^10^(1.00001, 1.00819, 0.10713) |  |

The cell parameters: *a* = 11.4826(12) Å, *b* = 13.4466(16) Å, *c* = 16.0371(18) Å , *V* = 2476.16(50) Å^3^ and *Z* = 4.

$$P_{s}=lim\frac{1}{V}\sum q_{i}r_{i}$$

$$=\frac{1.602*{10}^{-19}C*\left( 0.6650-0.3571 \right)*4*16.0371*{10}^{-10} m}{2476.16*{10}^{-30}m^{3}}$$

$$=0.12778 C m^{-2}$$

$$\left| P_{s} \right|=12.78 \mu C \mathrm{cm}^{-2}$$

Table S4. The $d_{33}$ values of six single crystals, their average value and error.

| Samples | 1 | 2 | 3 | 4 | 5 | 6 |
| --- | --- | --- | --- | --- | --- | --- |
| Piezoelectric coefficient (pC/N) | 66.0 | 68.0 | 71.6 | 61.9 | 73.6 | 63.4 |
| Average value | 67.42 | | | | | |
| Error | 1.87 | | | | | |

Table S5. The calculation of $g_{33}$ according to the equation: $g_{33}=\frac{d_{33}}{\varepsilon_{0}\times\varepsilon_{r}}$ .

| Frequency (Hz) | Dielectric permittivity | Piezoelectric coefficient (pC/N) | Piezoelectric voltage coefficient (10^-3^ V m/N) |
| --- | --- | --- | --- |
| 1000 | 2.75 |  | 2768.9 |
| 800 | 4.52035 |  | 1684.5 |
| 500 | 5.28754 |  | 1440.1 |
| 100 | 17.4577 | 67.42±1.87 | 436.2 |
| 10 | 24.8046 |  | 307.0 |
| 2 | 42.2087 |  | 180.4 |
| 1 | 72.4371 |  | 105.1 |

Table S6. The summary of *d*_33_, *g*_33_, FOM and *Q*_33_ for different material systems.

| Material systerm | Compound | *d*_33_  (pC/N) | *g*_33_  (10^-3^ V m/N) | FOM  (10^-15^ m^2^/N) | *Q*_33_  (m^4^/C^2^) | References |
| --- | --- | --- | --- | --- | --- | --- |
|  | DIPAB | 11 | 15.5 | 170.5 | 0.0337 | [[9](#_ENREF_10)] |
|  | triglycine sulfate (TGS) | 22 | 37.1 | 816.2 | - | [[9a](#_ENREF_10), [10](#_ENREF_12)] |
|  | Croconic acid (CA) | 5 | 47.1 | 235.5 | 0.1178 | [[9a](#_ENREF_10), [11](#_ENREF_14)] |
|  | ImClO_4_ | 41 | 77 | 3157 | 1.03 | [[12](#_ENREF_15)] |
|  | (AH)ReO_4_ | 90 | 495.84 | 44625.6 | 60.47 | [[13](#_ENREF_16)] |
|  | (3,3-DFCBA)Cl | 30.5 | 437.2 | 13334.6 | 4.29 | [[10b](#_ENREF_13)] |
| Metal-free | HOCH_2_(CF_2_)_3_CH_2_OH | 138 | 2450 | 338100 | 15.28 | [[14](#_ENREF_17)] |
| molecular | (HaaOH)BF_4_ | 22 | 165.7 | 3645.4 | 20.2 | [[15](#_ENREF_18)] |
| material | MDABCO-NH_4_-I_3_ | 14 | - | - | - | [[16](#_ENREF_19)] |
|  | NDABCO-NH_4_-Br_3_ | 63 | - | - | - | [[17](#_ENREF_21)] |
|  | [(CF_3_-C_6_H_4_-NH_3_)(18-crown-6)][TFSA] | 42 | 680 | 28560 | - | [[18](#_ENREF_23)] |
|  | [(NH_3_-TEMPO)(18-crown-6)][ClO_4_] | 17 | - | - | - | [[19](#_ENREF_24)] |
|  | [(NH_3_-TEMPO)(18-crown-6)][ReO_4_] | 13 | - | - | - | [[19](#_ENREF_24)] |
|  | [(Me_2_N(CH_2_)_2_NH_3_)(18-crown-6)][BF_4_] | 46.1 (*d*_22_) | 1000 (*g*_22_) | 46100 | - | [[20](#_ENREF_25)] |
|  | [(Histamine)(18-crown-6)_2_][BF_4_]_2_ | 5 | 112.94 | 564.7 | - | [[21](#_ENREF_26)] |
|  | [(CH_3_-C_6_H_10_-NH_3_)(18-crown-6)][ClO_4_] | 67.42 | 2768.9 | 186679.2 | 118.03 | **This work** |
|  | TMCM-MnCl_3_ | 185 | 1681 | 310985 | 21.01 | [[9a](#_ENREF_10)] |
|  | (TMCM)_2_SnCl_6_ | 137 | 980 | 134260 | 5.63 | [[22](#_ENREF_27)] |
| Metal-basic | TMBM-MnBr_3_ | 112 | 1120 | 125440 | 16 | [[23](#_ENREF_28)] |
| molecular | TMCM-CdBrCl_2_ | 440 | 6215 | 2724600 | 100.2 | [[24](#_ENREF_29)] |
| material | [C_6_H_5_N(CH_3_)_3_]CdBr_2_Cl_0.75_I_0.25_ | 367 | 3595 | 1222855 | 64.2 | [[25](#_ENREF_30)] |
|  | (TMFM)_0.26_(TMCM)_0.74_CdCl_3_ | 1540 | 9506 | 14639240 | 63.37 | [[26](#_ENREF_31)] |
|  | TMCM-GaCl_4_ | 226 | 1318 | 297868 | 10.3 | [[27](#_ENREF_32)] |
|  | (ATHP)_2_PbBr_4_ | 76 | 660.3 | 50160 | 5.9 | [[28](#_ENREF_33)] |
|  | PVDF | 33 | 310 | 10254 | -1.3 | [[29](#_ENREF_34)] |
| Polymers | P(VDF-TrFE) | 25 | 237 | 5934 | -1.5 | [[29b](#_ENREF_35), [30](#_ENREF_36)] |
|  | BOPVDF | 62 | 359 | 22274 | - | [[17a](#_ENREF_21)] |
| Inorganic | BaTiO_3_ | 191 | 11.37 | 2172 | 0.11 | [[31](#_ENREF_37)] |
|  | PbTiO_3_ | 143 | 129 | 19367 | 0.089 | [[32](#_ENREF_38)] |
|  | Pb(Zr_0.52_Ti_0.48_)O_3_ (PZT) | 220 | 34 | 7480 | - | [[33](#_ENREF_39)] |
|  | PZT-5A | 440 | 25 | 11000 | - | [[34](#_ENREF_40)] |
|  | KNN-ceramic | 275 | 30.26 | 8321.5 | - | [[35](#_ENREF_41)] |
|  | KNN-Li | 240 | 28.5 | 6840 | 0.016 | [[36](#_ENREF_42)] |
|  | SM-PT | 127 | 115 | 14605 | - | [[37](#_ENREF_43)] |
|  | CuInP_2_S_6_ | 100 | 272 | 27200 | 3.13 | [[38](#_ENREF_44)] |

Table S7. The summary of the output performance of the energy harvesting devices fabricated using composite films of different material systems.

| Material systerm | Composition | Active area  (cm^2^) | Force  (N) | Voltage  (V) | Current density  (*μ*A/cm^2^) | Power density  (*μ*W/cm^2^) | References |
| --- | --- | --- | --- | --- | --- | --- | --- |
|  | (HaaOH)BF_4_ | - | 31 | 5 | 0.31 μA | 1.2 | [[15](#_ENREF_18)] |
|  | DPDP∙PF_6_/PDMS | - | 15 | 8.5 | 0.5 μA | 1.74 μW/cm^3^ | [[39](#_ENREF_45)] |
| Metal-free | DPDP·BF_4_/TPU | 3 | 22 | 8.95 | 0.89 μA | 10.16 μW/cm3 | [[40](#_ENREF_46)] |
| molecular | MDABCO–NH_4_I_3_ | 2.25 | - | 15.9 | 54.5 nA | 0.2 | [[41](#_ENREF_47)] |
| material | *N*,*N*'-bis(4-nitrophenyl)methanediamine/PVA | - | 10 | 19.1 | 5.5 µA | 66 μW/cm^3^ | [[42](#_ENREF_48)] |
|  | [(*N*,*N*-dimethylethylenediammonium)(18-crown-6)][BF_4_] | 3.3 | 180 | 23 | - | 19.6 | [[20](#_ENREF_25)] |
|  | [(CH_3_-C_6_H_10_-NH_3_)(18-crown-6)][ClO_4_]/PDMS | 1 | 50 | 6 | 31 | 86.49 | **This work** |
|  | [(CH_3_-C_6_H_10_-NH_3_)(18-crown-6)][ClO_4_]/TPU | 1 | 150 | 120 | 6.1 | 432.1 | **This work** |
|  | PVDF | 8 | 8 | 10 | 6.2 | 0.81 | [[43](#_ENREF_49)] |
| Polymer | PVDF-TrFE shell/polycarbonate | - | - | 126 | 7.2 µA | 71 | [[44](#_ENREF_51)] |
|  | TMCM-GaCl_4_/PDMS | 4 | 1 | 38.1 | 0.4 | - | [[27](#_ENREF_32)] |
|  | (ATHP)_2_PbBr_2_Cl_2_/PDMS | 2 | 4.2 | 90 | 3.25 | 1.7 | [[45](#_ENREF_52)] |
| Metal-basic | TMCM_2_SnCl_6_/PDMS | 2 | 4.9 | 81 | 1 | 2.56 | [[22](#_ENREF_27)] |
| molecular | [RM3HQ]_2_RbPr(NO_3_)_6_/PU | 3 | 5 | 30 | 6 | 60 | [[46](#_ENREF_53)] |
| material | TMCM-CdCl_3_ micro-rods/PDMS | 1.21 | 5 | 41 | 3.45 | 115.2 | [[47](#_ENREF_54)] |
|  | TMCM-CdCl_3_/TPU | 1.78 | 80 | 103 | 23.6 | 636.9 | [[48](#_ENREF_55)] |
|  | HETMACdCl_3_/PDMS | 4 | 40 | 55.2 | 4.02 | 70.9 | [[49](#_ENREF_56)] |
|  | Gradient porous/PZT/PDMS | 30 | 100 | 152 | 0.5833 | 110 | [[50](#_ENREF_57)] |
|  | BT/PVDF | 6.25 | - | 11 | 0.216 | 4.1 | [[51](#_ENREF_58)] |
|  | BaTiO_3_/PDMS | 12.25 | - | 60 | 0.0898 | 3.27 | [[52](#_ENREF_59)] |
| Inorganics | BT/P(VDF-TrFE) | 6.25 | - | 12.6 | 0.208 | 0.68 | [[53](#_ENREF_60)] |
|  | PZT/PDMS | - | 8% stress | 85 | - (or 0.04μA) | - | [[54](#_ENREF_61)] |
|  | PZT/PVDF | 1.8 | - | 55 | - | 36 | [[55](#_ENREF_62)] |
|  | Ag/KNN heterostructure/PDMS | 8 | 80 | 240 | 2.875 | 141 | [[56](#_ENREF_63)] |
|  | Sm-PMN-PT/PVDF | 1.2 | 12 | 28 | 274.17 | - | [[57](#_ENREF_64)] |

**References**

[1] J. Zhang, T. Lu, *Phys. Chem. Chem. Phys.* **2021**, *23*, 20323-20328.

[2] a) Q. Gu, X. Lu, C. Chen, X. Wang, F. Kang, Y. Y. Li, Q. Xu, J. Lu, Y. Han, W. Qin, Q. Zhang, *Angew. Chem. Int. Ed.* **2024**, *63*, e202409708; b) R. A. Shaukat, Q. M. Saqib, J. Kim, H. Song, M. U. Khan, M. Y. Chougale, J. Bae, M. J. Choi, *Nano Energy* **2022**, *96*, 107128.

[3] M. Samet, V. Levchenko, G. Boiteux, G. Seytre, A. Kallel, A. Serghei, *J. Chem. Phys.* **2015**, *142*.

[4] G. Melepalliyalil, M. M. Mathew, S. Varghese, U. G. Panicker, *RSC Adv.* **2026**, *16*, 2271-2285.

[5] P. B. Ishai, M. S. Talary, A. Caduff, E. Levy, Y. Feldman, *Meas. Sci. Technol.* **2013**, *24*, 102001.

[6] E. Stojchevska, R. Popeski-Dimovski, Ž. Kokolanski, C. Gualandi, A. Bužarovska, *Macromol. Chem. Phys.* **2023**, *224*, 2200401.

[7] S. Paramee, R. Guo, A. S. Bhalla, H. Manuspiya, *Ferroelectrics* **2021**, *586*, 178-189.

[8] C. Chooseng, S. Chaipo, C. Putson, *J. Phys.: Conf. Ser.* **2021**, *2145*, 012043.

[9] a) Y.-M. You, W.-Q. Liao, D. Zhao, H.-Y. Ye, Y. Zhang, Q. Zhou, X. Niu, J. Wang, P.-F. Li, D.-W. Fu, Z. Wang, S. Gao, K. Yang, J.-M. Liu, J. Li, Y. Yan, R.-G. Xiong, *Science* **2017**, *357*, 306-309; b) D.-W. Fu, H.-L. Cai, Y. Liu, Q. Ye, W. Zhang, Y. Zhang, X.-Y. Chen, G. Giovannetti, M. Capone, J. Li, R.-G. Xiong, *Science* **2013**, *339*, 425-428.

[10] a) R.-G. Xiong, *Chin. Chem. Lett.* **2013**, *24*, 681-684; b) H.-Y. Zhang, *Chem. Sci.* **2022**, *13*, 5006-5013.

[11] S. Horiuchi, Y. Tokunaga, G. Giovannetti, S. Picozzi, H. Itoh, R. Shimano, R. Kumai, Y. Tokura, *Nature* **2010**, *463*, 789-792.

[12] Y. Zhang, Y. Liu, H.-Y. Ye, D.-W. Fu, W. Gao, H. Ma, Z. Liu, Y. Liu, W. Zhang, J. Li, G.-L. Yuan, R.-G. Xiong, *Angew. Chem. Int. Ed.* **2014**, *53*, 5064-5068.

[13] J. Harada, Y. Kawamura, Y. Takahashi, Y. Uemura, T. Hasegawa, H. Taniguchi, K. Maruyama, *J. Am. Chem. Soc.* **2019**, *141*, 9349-9357.

[14] H.-Y. Zhang, Y.-Y. Tang, Z.-X. Gu, P. Wang, X.-G. Chen, H.-P. Lv, P.-F. Li, Q. Jiang, N. Gu, S. Ren, R.-G. Xiong, *Science* **2024**, *383*, 1492-1498.

[15] Y.-A. Xiong, S.-S. Duan, H.-H. Hu, J. Yao, Q. Pan, T.-T. Sha, X. Wei, H.-R. Ji, J. Wu, Y.-M. You, *Nat. Commun.* **2024**, *15*, 4470.

[16] a) H.-Y. Ye, Y.-Y. Tang, P.-F. Li, W.-Q. Liao, J.-X. Gao, X.-N. Hua, H. Cai, P.-P. Shi, Y.-M. You, R.-G. Xiong, *Science* **2018**, *361*, 151-155; b) H. Wang, H. Liu, Z. Zhang, Z. Liu, Z. Lv, T. Li, W. Ju, H. Li, X. Cai, H. Han, *npj Comput. Mater.* **2019**, *5*, 17.

[17] a) Y. Huang, G. Rui, Q. Li, E. Allahyarov, R. Li, M. Fukuto, G.-J. Zhong, J.-Z. Xu, Z.-M. Li, P. L. Taylor, L. Zhu, *Nat. Commun.* **2021**, *12*, 675; b) H. Zhang, Z.-K. Xu, Z.-X. Wang, H. Yu, H.-P. Lv, P.-F. Li, W.-Q. Liao, R.-G. Xiong, *J. Am. Chem. Soc.* **2023**, *145*, 4892-4899.

[18] H.-P. Lv, Y.-R. Li, X.-J. Song, N. Zhang, R.-G. Xiong, H.-Y. Zhang, *J. Am. Chem. Soc.* **2023**, *145*, 3187-3195.

[19] C.-R. Huang, Y. Li, Y. Xie, Y. Du, H. Peng, Y.-L. Zeng, J.-C. Liu, R.-G. Xiong, *Angew. Chem. Int. Edit.* **2021**, *60*, 16668-16673.

[20] M.-M. Lun, J.-Q. Luo, Z.-X. Zhang, J. Li, L.-Y. Xie, H.-F. Lu, Y. Zhang, D.-W. Fu, *Chem. Eng. J.* **2023**, *475*, 145969.

[21] Y.-R. Li, Y.-F. Zhang, Y.-Y. Tang, H.-Y. Zhang, *Chem.l Commun.* **2022**, *58*, 5148-5151.

[22] G. Huang, A. A. Khan, M. M. Rana, C. Xu, S. Xu, R. Saritas, S. Zhang, E. Abdel-Rahmand, P. Turban, S. Ababou-Girard, C. Wang, D. Ban, *ACS Energy Lett.* **2020**, *6*, 16-23.

[23] W.-Q. Liao, Y.-Y. Tang, P.-F. Li, Y.-M. You, R.-G. Xiong, *J. Am. Chem. Soc.* **2017**, *139*, 18071-18077.

[24] X.-G. Chen, Y.-Y. Tang, H.-P. Lv, X.-J. Song, H. Peng, H. Yu, W.-Q. Liao, Y.-M. You, R.-G. Xiong, *J. Am. Chem. Soc.* **2023**, *145*, 1936-1944.

[25] Y. Hu, K. Parida, H. Zhang, X. Wang, Y. Li, X. Zhou, S. A. Morris, W. H. Liew, H. Wang, T. Li, F. Jiang, M. Yang, M. Alexe, Z. Du, C. L. Gan, K. Yao, B. Xu, P. S. Lee, H. J. Fan, *Nat. Commun.* **2022**, *13*, 5607.

[26] W.-Q. Liao, D. Zhao, Y.-Y. Tang, Y. Zhang, P.-F. Li, P.-P. Shi, X.-G. Chen, Y.-M. You, R.-G. Xiong, *Science* **2019**, *363*, 1206-1210.

[27] B. Wang, J. Hong, Y. Yang, H. Zhao, L. Long, L. Zheng, *Matter* **2022**, *5*, 1296-1304.

[28] X.-G. Chen, X.-J. Song, Z.-X. Zhang, P.-F. Li, J.-Z. Ge, Y.-Y. Tang, J.-X. Gao, W.-Y. Zhang, D.-W. Fu, Y.-M. You, R.-G. Xiong, *J. Am. Chem. Soc.* **2020**, *142*, 1077-1082.

[29] a) R. Xu, S.-G. Kim, *Fjgures of Merits of Piezoelectric Materials in Energy Harvesters*, **2012**; b) I. Katsouras, K. Asadi, M. Li, T. B. van Driel, K. S. Kjær, D. Zhao, T. Lenz, Y. Gu, P. W. M. Blom, D. Damjanovic, M. M. Nielsen, D. M. de Leeuw, *Nat. Mater.* **2016**, *15*, 78-84.

[30] Q. Sun, W. Xia, Y. Liu, P. Ren, X. Tian, T. Hu, *IEEE Transactions on Ultrasonics, Ferroelectrics, and Frequency Control* **2020**, *67*, 975-983.

[31] R. E. Newnham, L. J. Bowen, K. A. Klicker, L. E. Cross, *Mater. & Des.* **1980**, *2*, 93-106.

[32] T. Suwannasiri, A. Safari, *J. Am. Ceram. Soc.* **1993**, *76*, 3155-3158.

[33] C. A. Randall, N. Kim, J.-P. Kucera, W. Cao, T. R. Shrout, *J. Am. Ceram. Soc.* **1998**, *81*, 677-688.

[34] Y. Zhang, S. Wang, D. a. Liu, Q. Zhang, W. Wang, B. Ren, X. Zhao, H. Luo, *Sensors and Actuat. A: Phys.* **2011**, *168*, 223-228.

[35] B. Liu, P. Li, B. Shen, J. Zhai, Y. Zhang, F. Li, X. Liu, *J. Am. Ceram. Soc.* **2018**, *101*, 265-273.

[36] E. Hollenstein, M. Davis, D. Damjanovic, N. Setter, *Appl. Phys. Lett.* **2005**, *87*.

[37] Y. Yan, J. E. Zhou, D. Maurya, Y. U. Wang, S. Priya, *Nat. Commun.* **2016**, *7*, 13089.

[38] S. M. Neumayer, E. A. Eliseev, M. A. Susner, A. Tselev, B. J. Rodriguez, J. A. Brehm, S. T. Pantelides, G. Panchapakesan, S. Jesse, S. V. Kalinin, M. A. McGuire, A. N. Morozovska, P. Maksymovych, N. Balke, *Phys. Rev. Mater.* **2019**, *3*, 024401.

[39] T. Vijayakanth, A. K. Srivastava, F. Ram, P. Kulkarni, K. Shanmuganathan, B. Praveenkumar, R. Boomishankar, *Angew. Chem. Int. Ed.* **2018**, *57*, 9054-9058.

[40] T. Vijayakanth, F. Ram, B. Praveenkumar, K. Shanmuganathan, R. Boomishankar, *Chem. Mater.* **2019**, *31*, 5964-5972.

[41] H.-S. Wu, S.-M. Wei, S.-W. Chen, H.-C. Pan, W.-P. Pan, S.-M. Huang, M.-L. Tsai, P.-K. Yang, *Adv. Sci.***2022**, *9*, 2105974.

[42] S. Bhunia, S. K. Karan, R. Chowdhury, I. Ghosh, S. Saha, K. Das, A. Mondal, A. Nanda, B. B. Khatua, C. M. Reddy, *Chem* **2024**, *10*, 1741-1754.

[43] a) L. Lu, W. Ding, J. Liu, B. Yang, *Nano Energy* **2020**, *78*, 105251; b) K. Maity, S. Garain, K. Henkel, D. Schmeißer, D. Mandal, *ACS Appl. Polym. Mater.* **2020**, *2*, 862-878.

[44] B. Chai, K. Shi, Y. Wang, Y. Liu, F. Liu, P. Jiang, G. Sheng, S. Wang, P. Xu, X. Xu, X. Huang, *Nano Lett.* **2023**, *23*, 1810-1819.

[45] A. A. Khan, G. Huang, M. M. Rana, N. Mei, M. Biondi, S. Rassel, N. Tanguy, B. Sun, Z. Leonenko, N. Yan, C. Wang, S. Xu, D. Ban, *Nano Energy* **2021**, *86*, 106039.

[46] Q.-Q. Jia, H.-F. Lu, J.-Q. Luo, Y.-Y. Zhang, H.-F. Ni, F.-W. Zhang, J. Wang, D.-W. Fu, C.-F. Wang, Y. Zhang, *Small* **2024**, *20*, 2306989.

[47] Y.-J. Gong, Z.-G. Li, H. Chen, T.-M. Guo, F.-F. Gao, G.-J. Chen, Y. Zhang, Y.-M. You, W. Li, M. He, X.-H. Bu, J. Yu, *Matter* **2023**, *6*, 2066-2080.

[48] J.-Q. Luo, H.-F. Lu, Y.-J. Nie, Y.-H. Zhou, C.-F. Wang, Z.-X. Zhang, D.-W. Fu, Y. Zhang, *Nat. Commun.* **2024**, *15*, 8636.

[49] S. Deswal, S. K. Singh, R. Pandey, P. Nasa, D. Kabra, B. Praveenkumar, S. Ogale, R. Boomishankar, *Chem. Mater.* **2020**, *32*, 8333-8341.

[50] H. Liu, X. Lin, S. Zhang, Y. Huan, S. Huang, X. Cheng, *J. Mater. Chem. A* **2020**, *8*, 19631-19640.

[51] K. Shi, B. Sun, X. Huang, P. Jiang, *Nano Energy* **2018**, *52*, 153-162.

[52] C. Baek, J. H. Yun, H. S. Wang, J. E. Wang, H. Park, K.-I. Park, D. K. Kim, *Appl. Surf. Sci.* **2018**, *429*, 164-170.

[53] K. Shi, B. Chai, H. Zou, P. Shen, B. Sun, P. Jiang, Z. Shi, X. Huang, *Nano Energy* **2021**, *80*, 105515.

[54] G. Zhang, P. Zhao, X. Zhang, K. Han, T. Zhao, Y. Zhang, C. K. Jeong, S. Jiang, S. Zhang, Q. Wang, *Energy & Environ. Sci.* **2018**, *11*, 2046-2056.

[55] S. H. Wankhade, S. Tiwari, A. Gaur, P. Maiti, *Energy Rep.* **2020**, *6*, 358-364.

[56] Y. Huan, X. Zhang, J. Song, Y. Zhao, T. Wei, G. Zhang, X. Wang, *Nano Energy* **2018**, *50*, 62-69.

[57] L. Gu, J. Liu, N. Cui, Q. Xu, T. Du, L. Zhang, Z. Wang, C. Long, Y. Qin, *Nat. Commun.* **2020**, *11*, 1030.
